# The structural basis for high-affinity c-di-GMP binding to the GSPII-B domain of the traffic ATPase PilF from *Thermus thermophilus*

**DOI:** 10.1016/j.jbc.2024.108041

**Published:** 2024-11-29

**Authors:** Konstantin Neißner, Heiko Keller, Lennart Kirchner, Stefanie Düsterhus, Elke Duchardt-Ferner, Beate Averhoff, Jens Wöhnert

**Affiliations:** 1 Institute for Molecular Biosciences, Goethe-University Frankfurt/M., Frankfurt, Germany; 2 Center for Biomolecular Magnetic Resonance (BMRZ), Goethe-University Frankfurt/M., Frankfurt, Germany; 3 Molecular Microbiology and Bioenergetics, Institute for Molecular Biosciences, Goethe-University Frankfurt/M., Frankfurt, Germany

**Keywords:** protein structure, NMR-spectroscopy, cyclic-di-GMP, *Thermus thermophilus*, twitching motility, natural transformation, type IV pilus

## Abstract

c-di-GMP is an important second messenger in bacteria regulating, *for example* motility, biofilm formation, cell wall biosynthesis, infectivity, and natural transformability. It binds to a multitude of intracellular receptors. This includes proteins containing general secretory pathway II (GSPII) domains such as the N-terminal domain of the *Vibrio cholerae* ATPase MshE (MshEN) which binds c-di-GMP with two copies of a 24-amino acids sequence motif. The traffic ATPase PilF from *Thermus thermophilus* is important for type IV pilus biogenesis, twitching motility, surface attachment, and natural DNA-uptake and contains three consecutive homologous GPSII domains. We show that only two of these domains bind c-di-GMP and define the structural basis for the exceptional high affinity of the GSPII-B domain for c-di-GMP, which is 83-fold higher than that of the prototypical MshEN domain. Our work establishes an extended consensus sequence for the c-di-GMP–binding motif and highlights the role of hydrophobic residues for high-affinity recognition of c-di-GMP. Our structure is the first example for a c-di-GMP–binding domain not relying on arginine residues for ligand recognition. We also show that c-di-GMP–binding induces local unwinding of an α-helical turn as well as subdomain reorientation to reinforce intermolecular contacts between c-di-GMP and the C-terminal subdomain. Abolishing c-di-GMP binding to GSPII-B reduces twitching motility and surface attachment but not natural DNA-uptake. Overall, our work contributes to a better characterization of c-di-GMP binding in this class of effector domains, allows the prediction of high-affinity c-di-GMP–binding family members, and advances our understanding of the importance of c-di-GMP binding for T4P-related functions.

The acquisition of new genetic material is an important driving force of evolution enabling the adaptation to changing environmental conditions such as fluctuating pH-levels, temperature or salt concentrations, and the colonization of new ecological niches in many organisms. One of the most direct and thus fastest ways to acquire novel genetic material is the uptake of free DNA from the environment through sophisticated DNA-translocation machineries (1, 2). This process is called natural transformation (3). The thermophilic bacterium *Thermus thermophilus* has one of the most efficient and versatile DNA transport systems known so far (4, 5, 6). The DNA-translocation machinery of *T. thermophilus* consists of 15 different proteins that are clustered in the outer or inner membrane, the inter-membrane space, or the cytoplasm (2, 7). Overall, this multicomponent DNA-translocator complex consists of interconnected subcomplexes and extends as an integrated structure from the cytoplasm through both membranes and the periplasm into the extracellular environment (7, 8, 9, 10). Many of its components are also important for the biogenesis of the type IV pili (T4P) in *T. thermophilus* (2). These dynamic nanofibers mediate a coordinated multicellular movement called twitching motility which is caused by the external attachment and retraction of T4P. Moreover, T4P are important for surface adhesion and biofilm formation.

The assembly of both the T4P and the DNA transporter are powered by the cytoplasmic, hexameric traffic ATPase PilF. The latter interacts with the DNA transporter *via* PilM, forming the cytoplasmic face of the inner membrane platform consisting of PilMNO and PilC (7, 11, 12, 13). PilF is an assembly-ATPase from the PilB subfamily of the diverse AAA+-superfamily and forms a dumbbell-like hexameric complex. This complex can be structurally and functionally divided into N- and C-terminal halves that are connected by a stem-like structure (13, 14, 15, 16). The C-terminal part is a ring structure that consists of six ATPase domains each featuring a canonical Walker A and an atypical Walker B motif as well as a zinc-binding tetracysteine motif (11, 13, 16).

The N-terminal part of PilF harbors a remarkable arrangement of three successive general secretory pathway II (GSPII) domains (GSPII-A to GSPII-C) with high sequence similarity to each other. The presence of three consecutive GSPII domains at its N-terminus distinguishes PilF from other assembly ATPases as it has so far only been reported for PilF homologs in the *Deinococcus-Thermus* phylum (7). This sparks intriguing questions with regard to the function of such a triplicate arrangement of the GSP domains as well as to the contributions of the individual GSP domains to the function of PilF. The N-terminal half of the PilF hexamer is assumed to form a disk-like structure but recent cryo-EM structures of PilF were only resolved with low resolution and with an electron density corresponding only to two of the three GSP-domains for each PilF monomer in the hexameric complex (13, 16). Thus, detailed structural information is still missing for this part of PilF. Previous studies suggested that the GSPII domains are important for pilus assembly as well as for twitching motility but have no influence on ATPase activity (7).

Sequence comparisons among traffic ATPases revealed that the three GSPII domains of PilF are homologous to the N-terminal domain of the prototypic traffic ATPase MshE (MshEN) from *Vibrio cholerae* (17). In *V. cholerae*, MshE is required for the formation of mannose-sensitive haemagglutinin T4P needed for initial cell attachment and subsequent biofilm formation (18, 19, 20). MshEN specifically binds the universal bacterial second messenger cyclic dimeric guanosine monophosphate (c-di-GMP, Fig. S1) with an affinity of ∼500 nM, which is essential for biofilm formation in *V. cholerae* (17). A potentially c-di-GMP–binding MshEN-like domain is conserved in the N-terminus of many functionally equivalent traffic ATPases (17). Several traffic ATPases, such as PilB of *Myxococcus xanthus*, PilB of *Chloracidobacterium thermophilum*, and PilB2 of *Clostridium perfringens* were demonstrated to bind c-di-GMP (21, 22). In *M. xanthus*, c-di-GMP binding to PilB regulates its function in motility and biofilm formation (22), whereas in *C. thermophilum*, c-di-GMP binding to PilB was suggested to regulate exopolysaccharide production (23).

Co-crystallization studies of MshEN in complex with c-di-GMP revealed an unprecedented c-di-GMP–binding mode. The 145 residue-long MshEN domain can be divided into an N- and a C-terminal subdomain. The C-terminal subdomain is formed by three α-helices that surround a mixed three-stranded β-sheet and is connected *via* an inflexible linker to the N-terminal subdomain consisting of a tightly packed four-helix bundle (17). Importantly, the N-terminal subdomain of MshEN contains two copies of a 24 amino acids long sequence motif with the consensus RLGxx(L/V/I) (L/V/I)xxG(L/V/I) (L/V/I)xxxxLxxxxLxxQ. The two copies of this sequence motif are separated by a stretch of five amino acids. Each motif forms two α-helices and interacts with the two GMP moieties of c-di-GMP (17). Together, the two copies of the sequence motif fold into a 53-residue four-helix bundle which binds c-di-GMP in an elongated, slightly bulged conformation *via* extensive hydrophobic and cation-π stacking interactions that cover both planar faces of the two guanosine bases in c-di-GMP. In combination with a multitude of hydrogen-bonding interactions, c-di-GMP is bound tightly in a highly specific binding pocket (17). In detail, the sides of the two guanine bases facing the protein stack onto two triangular hydrophobic clusters each formed by three highly conserved leucine residues. The first leucine residue from each sequence motif forms a cluster with the last two leucine residues in the other motif (17). The other sides of the guanine bases facing away from MshEN are involved in cation- π-stacking interactions with the two arginine residues at the respective N-termini of the two motifs leaving the hydrophobic sides of the guanine bases shielded against the solvent. Both guanine bases are directly bound by two hydrogen bonds that involve the backbone amide protons of the conserved glycines at the third position of the consensus motif and the nonconserved fourth residues of each motif as the donor groups and the O6 and N7 atoms at the Hoogsteen edges of the guanine as acceptors, respectively (17). Additional hydrogen bonds from the ligand to the protein involve the ribose-phosphate backbone of c-di-GMP. For each phosphate group, one nonbridging oxygen forms a stable hydrogen bond with the backbone amide group of the conserved leucine at the second position and with the side chain amino group of the glutamine at the last position of each motif. Furthermore, the 2′-OH-groups of the respective ribose moieties form hydrogen bonds with the side chain oxygen atoms of the same glutamine residues. The amino group of the guanine base of one GMP moiety is involved in an additional intermolecular hydrogen bonding interaction with the sidechain of an aspartate residue in the C-terminal subdomain of MshEN. No other residues of the C-terminal subdomain are involved in contacts with the ligand (17).

Compared to the previously established c-di-GMP–binding modes of other bacterial c-di-GMP–binding domains such as PilZ (RxxxR and DxSxxG) (24, 25), GGDEF I-site (RxxD) (26), and degenerate EAL (ExLxR) (27) domains, which often bind c-di-GMP dimers, the MshEN c-di-GMP–binding mode is unique. In the previously described c-di-GMP–binding domains, the c-di-GMP–binding motifs generally feature intermolecular interactions centered around arginine residues (28). These arginine residues are involved in hydrogen bonding interactions with the Hoogsteen edges of the guanine bases or the ribose-phosphate ring of c-di-GMP as well as in cation-π stacking interactions with the guanine bases (28). In contrast, MshEN binds monomeric c-di-GMP and features unprecedented, extensive intermolecular hydrophobic stacking interactions in combination with arginine residues that are only involved in cation-π stacking as a potent c-di-GMP recognition mode (17). MshEN (K_D_ = 500 nM) belongs to the c-di-GMP–binding domains with the highest affinity to c-di-GMP reported so far. Its affinity is only exceeded by the PilZ domain of PlzD (K_D_ ∼ 100–300 nM) and the degenerate EAL-domain of FimX (K_D_ ∼ 100–200 nM) (24, 27). Thus, the extensive hydrophobic stacking interactions between MshEN and c-di-GMP are apparently a very important factor contributing to high-affinity ligand binding by this type of c-di-GMP–binding domain.

In a previous study, we reported on the sequence similarity of the GSPII A-C domains of PilF to MshEN and that each of the domains carries copies of the MshEN consensus motif sequence with varying degrees of homology (29, 30). Here, we investigate c-di-GMP binding to PilF by thermodynamically dissecting the contributions of the three GSPII domains of PilF to c-di-GMP binding by isothermal titration calorimetry (ITC)-measurements. Interestingly, we find that PilF binds only two molecules of c-di-GMP with different affinities and that the c-di-GMP–binding activities can be attributed to the GSPII-B and GSPII-C domains. Remarkably, the GSPII-B domain binds c-di-GMP with a K_D_ of only 6 nM and thus with an affinity ∼ 83fold higher than MshEN. GSPII-B exhibits the highest sequence similarity to MshEN. However, the two c-di-GMP–binding sequence motifs of GSPII-B differ in one important aspect from the consensus sequence defined from MshEN. In both GSPII-B motifs, the N-terminal arginine residues of the MshEN consensus sequence that form the above-mentioned stacking interactions with the guanine base moieties are replaced by either lysine or leucine. Thus, we investigated how these differences in c-di-GMP–binding motif sequences between the prototypical MshEN and the PilF GSPII-B domain affect the thermodynamics of the c-di-GMP interactions as well as their structural consequences. We present the tertiary structure of WT-GSPII-B in the apo and the c-di-GMP–bound state as well as the structures and ligand-binding properties of mutants with altered amino acids at key positions of the ligand-binding motif in the holo state. Thus, we further characterize the c-di-GMP–binding mode of MshEN-like domains and explore the exceptional high binding affinity capabilities of an extended MshEN consensus sequence. Remarkably, the PilF GSPII-B domain is the first structurally characterized c-di-GMP–binding domain where arginine residues are not involved in high-affinity ligand binding. Furthermore, we report that abolished c-di-GMP binding to the PilF GSPII-B domain affects T4P-related functions such as twitching motility and adhesion to solid surfaces.

## Results

### The three consecutive GSPII-domains in the PilF N-terminus bind two molecules of c-di-GMP with very different affinities

**Figure 1:**
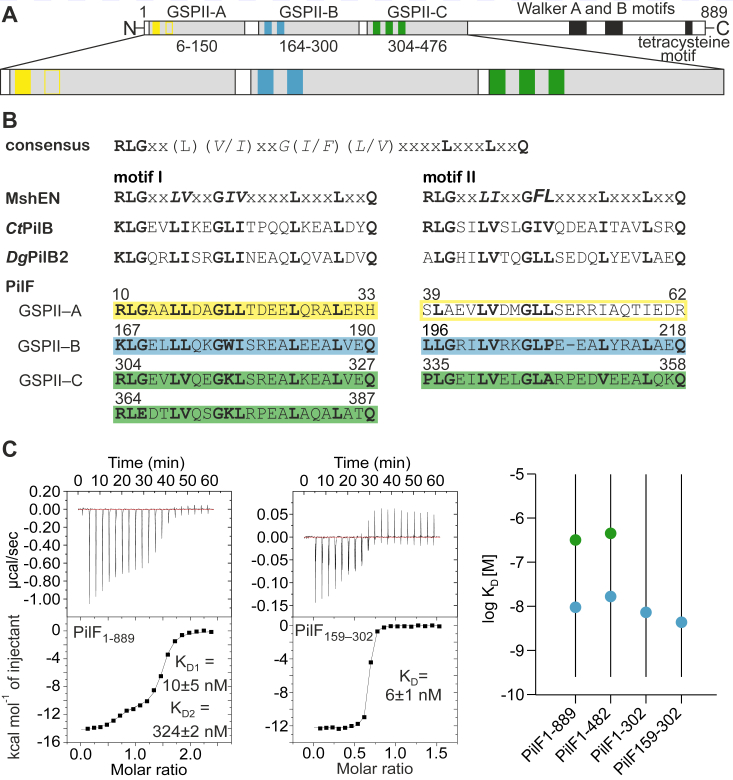
**Schematic overview of PilF domain structure and comparison of sequence and binding affinities for c-di-GMP-binding in PilF GSPII domains.***A*, cartoon representation of PilF with the three N-terminal GSPII domains GSPII-A, GSPII-B, and GSPII-C highlighted. The GSPII domains harbor one to three conserved copies of the 24-residue long c-di-GMP–binding sequence motifs as indicated by *yellow* (GSPII-A), *blue* (GSPII-B), and *green* (GSPII-C) bars, respectively. *B*, sequence comparison of the MshEN-derived c-di-GMP–binding consensus sequence motif to motifs in CtPilB (*Clostridium thermophilum*), DgPilB2 (*Deinococcus geothermalis*), and the PilF-GSPIIA-C domains. Colors are used as in *A*. *C*, (*left*) c-di-GMP–binding affinities for full length PilF and PilF_159-302_*via* isothermal titration calorimetry (ITC). (*right*) Graphical summary of K_D_ values for relevant PilF constructs.

While the PilT-class traffic ATPase PilF from *T. thermophilus* is an 889 amino acid long protein, only the C-terminal half (residues 483–889) is essential for ATP hydrolysis. This naturally raises the question of the function of the N-terminal half which contains three consecutive GSPII domains (GSPII-A – GSPII-C) spanning residues 6 to 150 (GSPII-A), 164 to 300 (GSPII-B), and 304 to 476 (GSPII-C) (Fig. 1*A*). All three GSPII domains harbor copies of MshEN-like c-di-GMP–binding sequence motifs with different degrees of conservation (17). The first GSPII domain (A) contains one nearly perfectly conserved and one degenerate motif, the second GSPII domain (B) two, and the third domain (C) even three conserved sequence motifs (Fig. 1*B*). While GSPII-A shows only one deviation from the consensus sequence in motif I at the last position (H33), motif II is only 45% identical to the consensus sequence in the key positions, so that overall GSPII-A shows the greatest deviation from the consensus sequence (Fig. 1*B*). In comparison, GSPII-C harbors three motifs homologous to the consensus sequence motif with the only deviation being the first residue of motif II (P335) (Fig. 1*B*). Both sequence motifs in the GSPII-B domain are highly similar to the consensus sequence (Fig. 1*B*). Among the three PilF GSPII domains, GSPII-B is closest to the MshEN consensus in sequence identity. However, a few features are unique to GSPII-B. First, the second motif from GSPII-B is one residue shorter than the consensus sequence as it lacks a glutamine residue (Q53 in MshEN). Second, the first residues in the two c-di-GMP–binding motifs of GSPII-B deviate from the consensus sequence. This residue is described to be a key residue in c-di-GMP binding of MshEN (17). While in MshEN, both motifs begin with arginine (R), these residues are replaced by lysine (K167) or leucine (L196) in the first and second motif of GSPII-B, respectively. Interestingly, lysine as the first residue in motif I is also present in other PilT-related ATPases that are expected to bind c-di-GMP such as *Ct*-PilB from *Chloracidobacterium thermophilum* or *Dg*-PilB2 from *Deinococcus geothermalis.* This indicates that certain alterations of the MshEN motif consensus sequence might be accepted while still allowing c-di-GMP binding (Fig. 1*B*) (17).

We thermodynamically characterized c-di-GMP binding to PilF using ITC for full-length PilF (PilF_1-889_) as well as for various domain truncation constructs (PilF_1-482_, PilF_159-482_, PilF_1-302_, PilF_159-302_). A full overview of all ITC titration experiments and the derived thermodynamic parameters is given in Table S1. Apparently, each full-length PilF_1-889_ monomer in the hexameric complex binds two c-di-GMP molecules (Fig. 1*C*) with the two very different affinities of 12 ± 5 nM (K_D1_) and 324 ± 2 nM (K_D2_). The C-terminally truncated construct PilF_1-482_ lacking the entire ATPase domain but containing all three N-terminal GSPII domains which is monomeric exhibits K_D_ values for c-di-GMP of 17 ± 1 nM (K_D1_) and 460 ± 36 nM (K_D2_) (Fig. S2). This indicates that the oligomeric state of PilF does not significantly influence its c-di-GMP–binding capabilities and that one of the GSPII domains is apparently not able to bind c-di-GMP (Figs. 1*C* and S2). In contrast, PilF_1-302_ which contains only the GSPII-A and the GSPII-B domains and PilF_159-302_ which contains only the GSPII-B domain bind only one c-di-GMP molecule with an exceptionally high affinity of 7 ± 2 nM and 6 ± 1 nM, respectively. Together, these data indicate that GSPII-A is the domain lacking the ability to bind c-di-GMP, whereas high affinity binding is attributed to the GSPII-B domain. Importantly, the high affinity of the GSPII-B domain for c-di-GMP is also preserved at higher temperatures since an ITC experiment at 45 °C revealed a K_D_ of 20 nM (Fig. S3). Additionally, there is obviously a drastic difference in the c-di-GMP–binding affinities for GSPII-B and -C. GSPII-C exhibits a c-di-GMP affinity that is similar to MshEN (K_D_ = 500 nM) while GSPII-B shows an 83-fold higher affinity than MshEN (17). The exceptionally high c-di-GMP–binding affinity of PilF_159-302_ (GSPII-B) combined with the two deviations from the consensus sequence at key positions (K167 and L196) prompted us to focus on PilF_159-302_ in order to determine the structural basis for the differences in the c-di-GMP–binding mode and affinity compared to MshEN. Furthermore, the similar c-di-GMP affinities measured for the individual domains in isolation or in the context of larger protein constructs (Fig. 1*C* and Table S1) argue against the presence of extensive cooperativity effects in c-di-GMP binding by PilF.

### The NMR solution and the crystal structure of PilF_159–302_ in complex with c-di-GMP

As shown in the preceding section, PilF_159-302_ binds c-di-GMP with an affinity ∼83-fold higher than MshEN in both the context of full-length PilF and the isolated GSPII-B domain. This corresponds to an increase in the free enthalpy of binding of ∼2.7 kcal/mol at 20 °C (17). The distinct deviations in the two sequence motifs constituting the c-di-GMP–binding site in PilF_159-302_ compared to the consensus sequence might be the leading cause for this drastic increase in binding affinity. The same sequence deviations from the MshEN-derived consensus are also observed in the putative c-di-GMP–binding motifs in PilT proteins from *C. thermophilum* and *D. geothermalis* (17, 23). Thus, a structural characterization of the role of these residues in c-di-GMP binding might contribute to an extension of the MshEN-derived consensus sequence and reveal correlations between sequence and c-di-GMP–binding affinity (17).

**Figure 2:**
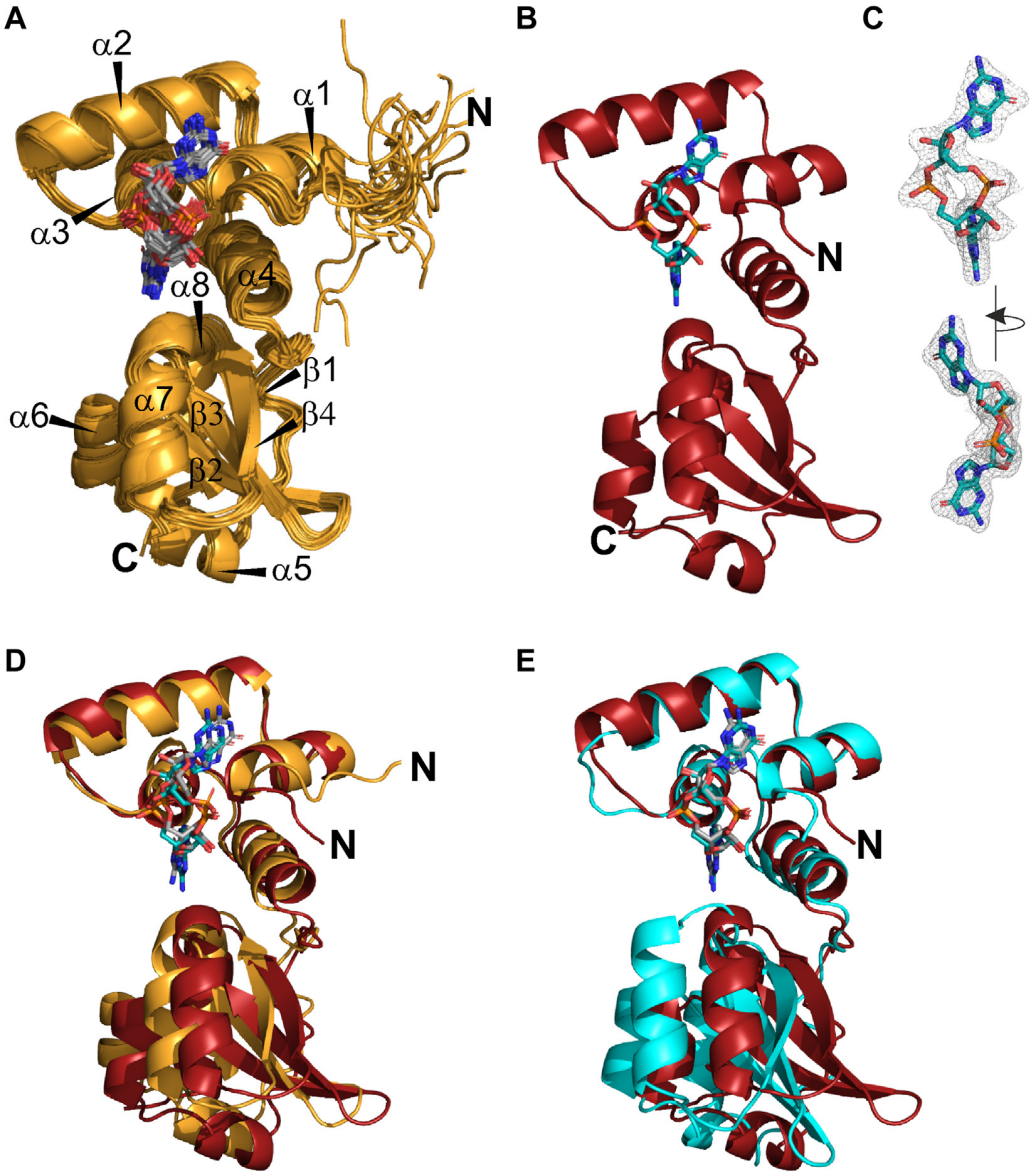
**Solution NMR and X-ray structures of PilF**_**159-302**_**in the c-di-GMP–bound state are similar to each other and to MshEN.***A*, NMR-solution structure bundle of PilF_159–302_ in the c-di-GMP–bound state. The 20 energy-minimized structures of PilF_159–302_ with the lowest CYANA target function are shown in cartoon representation and c-di-GMP in stick representation. *B*, X-ray structure of PilF_159–302_ (*cartoon*) with c-di-GMP (*sticks*) bound to the N-terminal subdomain in the same orientation as in (*A*). *C*, c-di-GMP in stick representation mapped into the well-defined F_o_-F_c_ difference electron density map of c-di-GMP in the PilF-c-di-GMP complex drawn at the 1.0 σ level. The good fit of c-di-GMP into the electron density map is representative for the crystal structure of PilF_159–302_ in complex with c-di-GMP. Two different orientations are shown. *D*, superposition of the lowest energy structure from (*A*) with the X-ray structure shown in (*B*). The alignment of both structures is based on the N-terminal subdomain. The C-terminal subdomains do not align very well due to slightly different subdomain orientation in the NMR and X-ray structures. *E*, superimposition of the N-terminal subdomains of the X-ray structures of PilF_159–302_ (*red*) and MshEN (*cyan*) in complex with c-di-GMP (17).

Therefore, we set out to gain detailed structural insights for PilF_159-302_ and its c-di-GMP–binding mode by solving the structure of PilF_159-302_ bound to c-di-GMP using NMR spectroscopy in solution and X-ray crystallography. The NMR structure calculations are based on thorough backbone and sidechain assignments presented previously (BMRB accession number 27853) (30). For PilF_159–302_ bound to c-di-GMP, we calculated a well-converged NMR structural bundle with an average backbone RMSD of 0.17 ± 0.07 Å and an average heavy atom RMSD of 0.62 ± 0.06 Å (Fig. 2*A* and Table 1) for the 20 lowest energy structures. This RMSD calculation excluded the flexible N- and C-terminal regions (159–166 and 300–302). Structures are derived form 3492 nuclear Overhauser effect (NOE) distance restraints and 226 backbone dihedral angles with the addition of four directly and two indirectly detected hydrogen bonds between c-di-GMP and protein backbone residues. Furthermore, the ribose-sugar pucker was determined to be in the C3′-endo conformation for both ribose moieties of the bound c-di-GMP according to the HCCH-TOCSY-E.COSY spectrum (Fig. S4).

**Table 1:** Statistics for NMR structure calculations and refinement

Conformational restricting restraints	PilF_159–302_ holo	PilF_159–302_ apo
Total NOE distance restraints	3492	2816
Intraresidue, |*i-j*| = 0	703	639
Sequential, |*i-j* | = 1	882	711
Short range, |*i-j*| < = 1	1585	1350
Medium range, 1< |*i-j*| <5	825	618
Long range, |*i-j*| > = 5	1082	848
Dihedral angle restraints (Talos+)	226	232
No. of restraints per residue	25.5	20.9
Residual restraint violations		
Average no. of distance violations per structure		
0.1–0.2 Å	0	0
0.2–0.5 Å	0	0
> 0.5 Å	0	0
Average no. of dihedral angle violations per structure		
1–10°	0	0
>10°	0	0
Model quality (ordered residues)		
Structures in final ensemble	20	20
Target function value	2.36 ± 0.04	1.09 ± 0.04
R.m.s deviation backbone atoms (Å)	0.17 ± 0.07	0.45 ± 0.19
R.m.s deviation heavy atoms (Å)	0.62 ± 0.06	0.89 ± 0.12
MolProbity Ramachandran statistics		
Most favored regions	89.2	89.6
Allowed regions	10.8	10.4
Disallowed regions	0	0
Model contents		
Ordered residue ranges (hetNOE > 0.6)	167–300	165–300
Total No. of residues	146	146
BMRB accession number	27,853	27,852
PDB code	8PKZ	8PQU

The crystal structure of PilF_159–302_ in complex with c-di-GMP was determined by molecular replacement using the NMR-solution structure of c-di-GMP–bound PilF_159–302_ as a starting point (Fig. 2*B*). Interestingly, MshEN or its domains as template did not lead to viable molecular replacement solutions as the solutions displayed a low TF Z-score (5.7) and overall high r-free (0.5252) values (31, 32). The crystals diffracted to ∼2 Å and the protein chain could be traced without interruption from residue 165 to 301 with c-di-GMP fitting perfectly into the difference electron density (Fig. 2*C* and Table 2). Based on the Ramachandran plot, all residues are in the most favored region and side chain rotamer outliers could not be detected. As already observed for the crystal structure of MshEN (5htl), the unit cell contains two copies of the protein with a highly similar overall structure (Table S2 and Fig. S5). Since for both the crystals of MshEN and PilF_159–302_ as well as for the PilF_159–302_ variants the electron density in the vicinity of the bound ligand is better defined in chain A than in chain B (Fig. S6), we refer in our structure comparisons to chain A if not explicitly mentioned otherwise. However, an overview of all pairwise RMSD values for all chains in all protein variants on the level of the full-length protein or the respective subdomains is given in Tables S2–S4.

**Table 2:** Statistics of data collection and refinement for X-ray datasets

	WT-PilF_159-302_	PilF_159-302_ K167R	PilF_159-302_ K167L
Data collection			
Wavelength (Å)	1	0.976	0.976
Resolution range	41.33-2.0 (2.071-2.0)	39.62-1.8 (1.864-1.8)	41.44-1.9 (1.968-1.9)
Space group	R3H	R3H	R3H
Unit cell	108.138 108.138 87.922 90 90 120	108.165 108.165 87.446 90 90 120	108.434 108.434 88.125 90 90 120
Total reflections	135,076 (13,119)	365,005 (37,373)	316,941 (32,171)
Unique reflections	25,934 (2624)	35,354 (3588)	30,444 (3034)
Multiplicity	5.2 (5.0)	10.3 (10.4)	10.4 (10.6)
Completeness (%)	99.96 (99.96)	99.94 (100.00)	99.96 (99.93)
Mean I/sigma(I)	29.09 (2.60)	18.04 (1.40)	15.02 (1.23)
Wilson B-factor	37.88	43.75	44.6
R-merge	0.03878 (0.6646)	0.05805 (1.393)	0.07163 (1.713)
R-meas	0.04317 (0.7432)	0.06116 (1.465)	0.07526 (1.8)
R-pim	0.01886 (0.3309)	0.01905 (0.4531)	0.02302 (0.5516)
CC1/2	1 (0.779)	0.998 (0.737)	0.999 (0.635)
CC∗	1 (0.936)	1 (0.921)	1 (0.881)
Refinement			
Reflections used	25,928	35,349	30,436
Reflections used for R-free	755	1774	1629
R-work	0.1791	0.1893	0.1796
R-free	0.2114	0.2238	0.2203
CC(work)	0.966	0.963	0.968
CC(free)	0.942	0.959	0.963
Number of nonhydrogen atoms	2487	2371	2467
Macromolecules	2130	2113	2157
Ligands	179	162	179
Solvent	178	96	131
Protein residues	276	275	283
RMS(bonds)	0.008	0.007	0.007
RMS(angles)	0.81	0.82	0.8
Ramachandran favored (%)	100	100	100
Ramachandran allowed (%)	0	0	0
Ramachandran outliers (%)	0	0	0
Rotamer outliers (%)	0	0	0
Clashscore	3.32	3.6	4.14
Average B-factor	46.62	57.32	57.18
Macromolecules	44.75	56.02	55.66
Ligands	61.84	74.02	73.22
Solvent	53.74	57.64	60.34

The NMR- and X-ray structures of PilF_159-302_ in the c-di-GMP–bound state are very similar to each other and both can be partitioned into an N- and a C-terminal subdomain that are connected by an inflexible linker (residues 222–233) (Fig. 2, *A* and *B*). The N-terminal subdomain spans residues 167 to 221 and consists of a tightly packed four helix bundle in which helix α1 and α2 as well as α3 and α4 are parallel to each other. The C-terminal subdomain exhibits a fold in which a mixed four-stranded β-sheet at the core of the subdomain is surrounded by four helices and the inflexible linker (Fig. 2, *A* and *B*). Only helix α4 of the N-terminal subdomain is in close contact with the C-terminal subdomain. In sum, the PilF_159-302_ structure consists of eight α-helices and four β-strands which is consistent with the previously derived secondary structure (30). c-di-GMP is bound in an elongated, slightly bulged conformation to the N-terminal subdomain with only the amino group of one of the guanine bases oriented towards the C-terminal subdomain (Fig. 2, *A*–*C*). Thus, the majority of protein–ligand interactions involve residues in the N-terminal subdomain, which is consistent with c-di-GMP binding to MshEN.

Superposition of the rigid portions (167–300) of the NMR structure with the lowest target function and the X-ray structure of PilF_159-302_ in complex with c-di-GMP yields a backbone RMSD of ∼ 1.3 Å showing the high similarity of the structures in solution and in the crystal. By only superimposing the N-terminal subdomains (167–221) in the two structures (RMSD ∼ 1 Å), a small difference in the relative orientation of the two subdomains with respect to each other in the solution and the crystal structure becomes evident (Fig. 2*D*). One likely cause for the difference in relative orientation are crystal packing forces. The superimposition of the C-terminal subdomains from the solution and the crystal structure yields a backbone RMSD of ∼ 0.7 Å (Fig. S7).

PilF_159-302_ in the c-di-GMP–bound state is very similar to MshEN with regard to its secondary structure and its overall topology. The overall backbone RMSD of the rigid parts (167–300 for PilF and 9–141 for MshEN) between the X-ray structures of the two proteins is only ∼ 1.3 Å. Notable differences are a C-terminally extended helix α2 in the N-terminal subdomain and the presence of two additional secondary structure elements of PilF_159-302_ in comparison to MshEN (17). In the linker connecting the subdomains, a short β-sheet (aa 223–225) runs parallel to β4, and in the C-terminal subdomain, an additional helix α5 is present at the bottom of the domain. Differences in the relative orientation of the two subdomains between the two proteins become evident by superimposing only their N-terminal subdomains (RMSD ∼ 0.8 Å, Fig. 2*E*). Superimposing the C-terminal subdomains leads to an RMSD value of 1.7 Å, which is the result of different placements of helices 7 and 8 relative to the central β-sheet between the structures of PilF_159-302_ and MshEN.

Overall, the location and the orientation of the c-di-GMP molecules in the N-terminal subdomains of both proteins are very similar, thus yielding no obvious explanation for the drastically increased affinity of PilF_159–302_ to c-di-GMP compared to MshEN (Fig. 2*E*) (17).

### Intermolecular hydrogen bonds between the PilF_159-302_ N-terminal subdomain and c-di-GMP

Comparing the tertiary structures of PilF_159-302_ and MshEN in the c-di-GMP–bound state does not reveal any major conformational differences that could rationalize the drastic divergence in the binding affinities to c-di-GMP. Thus, a thorough characterization of the PilF_159-302_ c-di-GMP–binding mode was imperative with a special focus on intermolecular hydrogen bonds and stacking interactions in comparison to MshEN (17).

**Figure 3:**
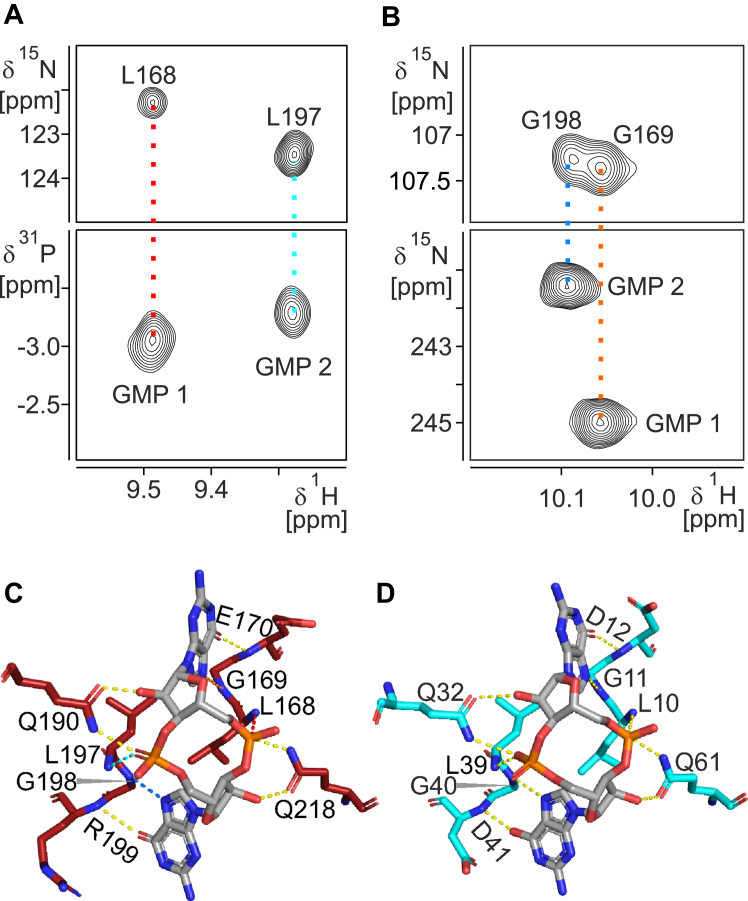
**Hydrogen bonds between the PilF**_**159–302**_**N-terminal subdomain and c-di-GMP.***A*, 2D-^1^H,^31^P-SOFAST-HMQC and (*B*) 2D-HNN-COSY spectra of PilF_159–302_ in complex with c-di-GMP for the direct detection of protein-ligand hydrogen bonds. The ^1^H chemical shift values of the respective ^1^H,^31^P or ^1^H,^15^N cross-peaks for bound c-di-GMP are compared to the ^1^H,^15^N-cross-peaks of the protein backbone amide groups in a 2D-^15^N-HSQC spectrum (*top*) to identify hydrogen bonding. The colors of the dotted lines connecting the cross-peaks with the identical proton chemical shift represent the hydrogen bonds in the three-dimensional structure shown in (*C*). *C*, intermolecular hydrogen bonds of holo PilF_159-302_ between the Hoogsteen edges and the ribose-phosphate backbone of c-di-GMP and protein backbone amide protons as well as side chain amino groups and oxygen atoms. *D*, intermolecular hydrogen bonds of MshEN in complex with c-di-GMP as deduced from the X-ray structure (pdb: 5htl).

Through dedicated NMR experiments optimized for magnetization transfer across potential hydrogen bonds, we directly detected four intermolecular hydrogen bonds between PilF_159-302_ and c-di-GMP (Fig. 3, *A* and *B*). A 2D-^1^H,^31^P-SOFAST-HMQC spectrum of c-di-GMP bound to PilF_159-302_ in combination with a standard ^15^N-HSQC of the amide region revealed two HP-correlation signals corresponding to two hydrogen bonds involving the c-di-GMP phosphate groups and the backbone amide groups of L168 and L197 (Fig. 3*A*). Additional intermolecular hydrogen bonds were directly detected in a 2D-BEST-TROSY-HNN-COSY spectrum that revealed correlations between the backbone amide proton resonances of either G169 or G198 and the N7 nitrogen atoms at the Hoogsteen edges of the two guanine-moieties of bound c-di-GMP (Fig. 3*B*). Both hydrogen bonds are also implied in the X-ray structure of holo PilF_159-302_ as indicated by the distance and angles of the respective atoms to one another (Fig. 3*C*). Furthermore, close inspection of the c-di-GMP–binding pocket in the X-ray structure indicated the presence of six additional direct intermolecular hydrogen bonds (Fig. 3*C*). Thus, for the GMP moieties of c-di-GMP, the O6 atoms are in close proximity to the backbone amide groups of the third residue in motif I (E170) and motif II (R199), respectively, indicating hydrogen bonding between these partners (Fig. 3*C*). Additionally, the amino groups and oxygen atoms of Q190 and Q218 side chains are oriented towards the ribose-phosphate backbone of c-di-GMP, where they form hydrogen bonds with nonbridging phosphate oxygens and the ribose 2′-OH groups, respectively (Fig. 3*C*). The presence of these hydrogen bonds in solution is also consistent with our NMR-data. There, the amide groups of E170 and R199 as well as the amino groups of Q190 and Q218 all exhibit extreme chemical shifts and/or significant chemical shift perturbations upon c-di-GMP binding (Fig. S8). In addition, the E170 and R199 H^N^ resonances are shifted upfield to the far right border of the ^15^N-HSQC spectrum compared to canonical H^N^ resonances (Fig. S8). The far downfield chemical shifts for the amino group resonances of Q190 and Q218 are very different from the canonical region of amino group chemical shifts (Fig. S8).

The X-ray structure of PilF_159-302_ bound to c-di-GMP suggests that two water-mediated hydrogen bonds involving the Y212 side chain hydroxyl group and one guanine amino group as well as the side chain of K219 and a phosphate group of the ligand might augment the set of 10 direct intermolecular hydrogen bonds. However, these water-mediated hydrogen bonds are only observed for chain A in the unit cell and might therefore not be very stable (Fig. S9, *A* and *B*).

The 10 direct intermolecular hydrogen bonds identified between the N-terminal subdomain of PilF_159-302_ and c-di-GMP are all conserved in comparison to the intermolecular hydrogen bonds described for MshEN (Fig. 3*D*), highlighting the importance of these conserved residues for the MshEN-like c-di-GMP–binding mode (17). While MshEN binds with a lower affinity to c-di-GMP than PilF159 to 302, it even features a direct intermolecular hydrogen bond not present in the PilF_159-302_-c-di-GMP complex between the side chain of an arginine preceding the first consensus motif and a backbone phosphate group of the ligand (Fig. S9, *C* and *D*). This residue is an aspartate in PilF_159-302_ thereby preventing the formation of an equivalent hydrogen bond at this position. Furthermore, the two complexes deviate in the number and the position of water-mediated intermolecular hydrogen bonds but these differ also between chain A and B in the structure of MshEN (Fig. S9).

### Hydrophobic stacking interactions between c-di-GMP and residues in the N-terminal subdomain

**Figure 4:**
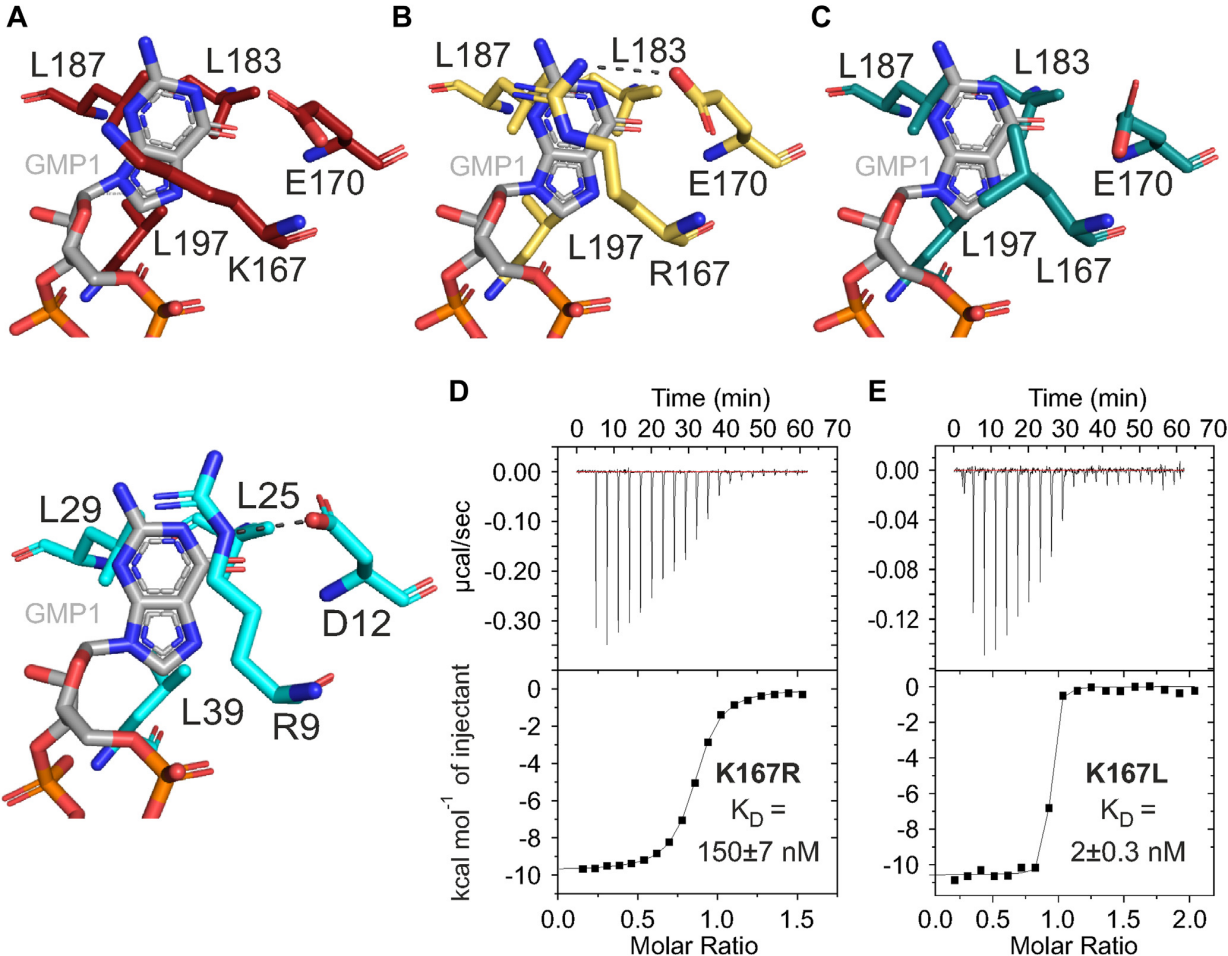
**The MshEN consensus sequence can be extended**. *A*, in WT PilF_159-302_ (*red*, top), K167 stacks on the guanine base of GMP1 in a hydrophobic interaction. In MshEN (*cyan*, *bottom*), R9 is involved in a cation-π-stacking interaction with GMP1 (17). *B*, the X-ray structure of the PilF_159-302_ K167R mutant shows R167 involved in a cation-π-stacking interaction with GMP1 with the R167 side chain held in place by hydrogen bonding to the E170 side chain. *C*, the X-ray structure of the PilF_159-302_ K167L mutant shows L167 involved in an extensive hydrophobic stacking interaction with GMP1. *D* and *E*, c-di-GMP–binding assays of PilF_159-302_ K167R (*D*) and PilF_159-302_ K167L (*E*) using ITC.

Further, we focused on the PilF_159-302_ c-di-GMP–binding pocket with the emphasis on possible guanine base stacking interactions as described previously for MshEN (17). Together, both c-di-GMP–binding motifs form two triangular hydrophobic clusters in the N-terminal subdomain. Each hydrophobic cluster involves three highly conserved leucine residues that are distributed along both c-di-GMP–binding motifs (Fig. S10). The N-terminal hydrophobic cluster is formed by L183 and L187 from motif I and L197 from motif II while L168 from motif I and L211 and L215 from motif II form the C-terminal hydrophobic cluster (Fig. S10). For both hydrophobic clusters, the leucine side chain methyl groups stack on top of the respective c-di-GMP guanine bases (Fig. S10, *A* and *B*). This in turn is identical to the binding mode observed for MshEN (17). There, the surfaces of the guanine bases that are not involved in the leucine stacking interaction are involved in cation-π-stacking interactions with the guanidinium groups of the arginine residues at the start of both motifs tucking in the guanine bases (17). In contrast to MshEN, no arginines are found in PilF_159-302_ at the corresponding positions. Instead, K167 and L196 are located at these positions (Fig. 1*B*). Inspection of the presented X-ray and NMR-structures of PilF_159-302_ in complex with c-di-GMP shows that these two residues are involved in extensive hydrophobic stacking interactions with the two guanine bases (Figs. 4*A*, S6*B*, and S11). The sidechain of K167 extends diagonally across the GMP1 guanine base plane with its hydrophobic region (Cβ to Cε methylene groups) located on top of the guanine base (Figs. 4*A*, S6*B*, and S11). Furthermore, the cationic amino group of K167 is in a position enabling a cation-π-interaction in addition to the hydrophobic stacking. While the exact positioning of the K167 side chain differs between the two chains in the crystal structure, both K167 conformations allow the hydrophobic stacking and the cation-π-interaction (Fig. S11). For L196, both methyl groups stack on the guanine base of GMP2 (Figs. S10, *C* and *D* and S11).

These deviations from the MshEN-binding mode prompted us to investigate the thermodynamic contribution of these residues to the extremely high affinity of PilF_159-302_ for c-di-GMP. Initially, we introduced arginine residues at these positions to reestablish an MshEN-like binding mode. ITC measurements with PilF_159-302_ K167R and PilF_159-302_ L196R revealed a substantial c-di-GMP affinity loss for both constructs (Figs. 4*D* and S12). We found a 25-fold higher K_D_ value of 150 ± 7 nM for K167R compared to the WT (6 nM) and a 4-fold higher K_D_ value of 23 ± 2 nM for L196R (Figs. 4*D* and S12). The PilF_159-302_ K167R variant crystallized well and diffracted up to 1.8 Å with the macromolecular chain being traceable from residue 165 to 301 without interruption. No Ramachandran or sidechain outliers were detected (Table 2). The side chain of R167 is well resolved in chain A in the asymmetric unit (Figs. S6*C* and S13). The structure of chain A showed that in this mutant R167 is indeed involved in a cation-π-stacking interaction with the GMP1 guanine base (Fig. 4*B*). However, only partial electron density is observed for the side chain of R167 and the neighboring E170 in chain B of the crystal structure (Fig. S6*C*) and there the R167 side chain guanidine group appears to be oriented towards a phosphate group of the ligand (Fig. S6*C*). Thus, the R167 stacking interaction with the guanine base of the ligand seems to be more dynamic and less energetically favorable than the equivalent interaction in the WT. In comparison to MshEN (chain A), the arginine side chain in chain A is positioned more towards the center of the guanine base (Figs. 4, *A* and *B* and S14) (17). This is most likely due to the presence of E170, which positions R167 by forming simultaneous hydrogen bonds with its backbone amide and the guanidinium group of its side chain (Fig. 4*B*). In MshEN, the amino acid corresponding to E170 is an aspartate whose side chain is shorter than in glutamate and hydrogen bonds only to the guanidinium group of the arginine (17). This leads to a different position of the R9 arginine side chain relative to the guanine base in MshEN (Figs. 4, *A*, bottom, and *B*, and S14*A*). In order to test if the large difference in binding affinity between the WT PilF_159-302_ and the K167R mutant is due to the actual amino acid replacement or the difference in the orientation of the arginine side chain in the K167R mutant caused by E170, we created the PilF_159-302_ K167R-E170D double mutant to mimic the side chain arrangement found in MshEN. ITC measurements with this construct and c-di-GMP revealed a K_D_ value of 92 ± 8 nM (Fig. S14*B*), which is similar to the K_D_ of c-di-GMP binding for the K167R single mutant.

The loss of binding affinity for PilF_159-302_ K167R in combination with the presence of a more dynamic cation-π-stacking interaction of R167 on the guanine base of GMP1 indicate that a lysine side chain at this position is beneficial for c-di-GMP binding most likely due to the presence of more extensive hydrophobic interactions. This represents the first difference of a PilF GSPII domain to MshEN that helps to rationalize the higher affinity of PilF_159-302_ to c-di-GMP (17). It is intriguing that other c-di-GMP–binding proteins with MshEN-like domains such as PilB2 from *D. geothermalis* and PilB from *C. thermophilum* also harbor lysine residues corresponding to K167 of PilF_159-302_ (17, 23) and have K_D_ values of 13.5 and 53 nM, respectively, that are also significantly lower than those observed for the prototypical MshEN domain.

To further test if in general a hydrophobic interaction is indeed beneficial to high affinity c-di-GMP binding or if this effect is lysine specific, we created the K167L PilF_159-302_ variant and characterized its c-di-GMP–binding affinity (Fig. 4, *C* and *E*). Remarkably, the ITC measurement with PilF_159-302_ K167L yielded an even lower K_D_ value of 2 ± 0.3 nM than WT-PilF_159-302_ showing that the large hydrophobic side chain L167 might improve c-di-GMP binding of PilF_159-302_ (Fig. 4, *C* and *E*) even further.

Thus, we crystallized the PilF_159-302_ K167L variant, which yielded crystals diffracting to 1.9 Å with the protein chain being traceable from residue 161 to 301 with no Ramachandran plot outliers (Fig. S13, *B* and *D*). The L167 leucine side chain is well resolved and in a similar conformation in both chains of the asymmetric unit (Fig. S6*D*) and involved in an extensive hydrophobic stacking interaction with the c-di-GMP guanine base (Figs. 4*C* and S13*D*). Combining the data from WT-PilF_159-302_ together with K167R and K167L variants compared to MshEN and as well as with *Dg*PilB2 and *Ct*PilB shows that the MshEN consensus sequence RLGxx(*L*/*V*/*I*) (*L*/*V*/*I*)xx*G*(*L*/*V*/*I*) (*L*/*V*/*I*)xxxxLxxxxLxxQ for c-di-GMP binding can be extended to (R/K/L)LGxx(*L*/*V*/*I*) (*L*/*V*/*I*)xx*G*(*L*/*V*/*I*) (*L*/*V*/*I*)xxxxLxxxxLxxQ. Moreover, a large hydrophobic residue at the very first position of the c-di-GMP–binding motif is apparently thermodynamically and dynamically beneficial for very high affinity c-di-GMP binding.

### The C-terminal subdomain and its role in c-di-GMP binding

**Figure 5:**
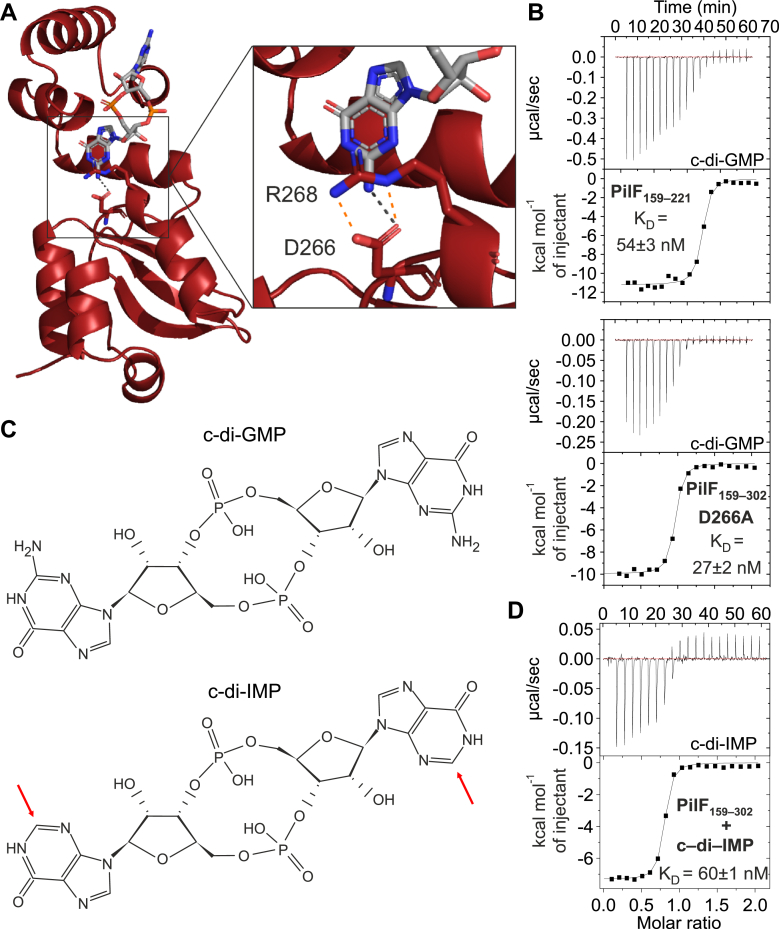
**The role of the PilF**_**159-302**_**C-terminal subdomain in c-di-GMP binding.***A*, X-ray structure of PilF_159–302_ (*red*) in complex with c-di-GMP. PilF_159-302_ is shown in a cartoon representation. The sidechains of residues D266 and R268 are depicted as *sticks*. The *black dotted line* indicates a hydrogen bond between one Oδ of D266 and the amino group of GMP2. Possible hydrogen bonds between the guanidinium group of R268 and the D266 oxygen atoms are depicted as *orange* dotted lines. *B*, ITC measurements for investigating the importance of the C-terminal subdomain for c-di-GMP binding. The thermograms and binding isotherms show c-di-GMP binding to PilF_159-221_ (*top*) and to PilF_159-302_ D266A (*bottom*). *C*, chemical structures of c-di-GMP (*top*) and c-di-IMP (*bottom*). The *red* arrows highlight the absence of the amino groups in c-di-IMP. *D*, ITC thermogram (*top*) and binding isotherm (*bottom*) of PilF_159-302_ binding to c-di-IMP.

While interactions of PilF_159-302_ with c-di-GMP described in the preceding sections were restricted to the N-terminal subdomain (PilF_159–221_), a polar interaction is found between c-di-GMP and the C-terminal subdomain between one Oδ of residue D266 and the H_2_N group at the Watson-Crick-edge of the GMP2 nucleobase (Figs. 5*A* and S9, *A* and *B*) in the X-ray structure. Interestingly, the sidechain of R268 with its guanidinium group forms two salt bridges with the D266 side chain (Fig. 5*A*). This positions the D266 sidechain in an orientation that favors the formation of the hydrogen bond to the amino group of GMP2 (Fig. 5*A*).

A similar polar interaction has been postulated for MshEN between an aspartate (D108) equivalent to D266 in PilF and the amino group of c-di-GMP. However, the arginine residue equivalent to R268 of PilF_159-302_ to orient the aspartate side chain is missing in MshEN (17). Furthermore, in MshEN, this interaction is present only in chain A of the unit cell (Fig. S9, *C* and *D*). In chain B, the D108 side chain interacts with an arginine from the N-terminal subdomain (R38) which is thereby sequestered from taking part in intermolecular stacking interactions with one of the guanine bases (Fig. S9, *C* and *D*). Interestingly, in MshEN, the removal of the interaction between the ligand and the C-terminal subdomain by deleting the entire C-terminal subdomain (17) leads to a slight increase in ligand affinity (350 nM compared to the 500 nM of full length MshEN) (17), suggesting that this interaction is not beneficial for ligand binding in MshEN. However, ITC-measurements for PilF_159–221_ lacking the C-terminal subdomain with c-di-GMP show an opposite effect. PilF_159-221_ binds c-di-GMP with a K_D_ value of 54 ± 3 nM that is 9-fold higher than the K_D_ value for PilF_159-302_ (6 nM) (Fig. 5*B*). While in MshEN, the C-terminal subdomain might have no or even a slightly hindering effect on c-di-GMP binding, the C-terminal subdomain is indeed beneficial for c-di-GMP binding in the context of PilF_159-302_. Interestingly, this is consistent with the extension of helix α7 of the C-terminal subdomain in PilF_159-302_ and the different relative orientation of the two subdomains in PilF_159-302_ compared to MshEN which leads to a different orientation of the C-terminal subdomain towards the c-di-GMP–binding site. To further test this hypothesis, we created PilF_159-302_ D266A in which the above-described hydrogen bond from the C-terminal subdomain to c-di-GMP is removed. A K_D_ value of 27 ± 2 nM that is similar to the PilF_159-221_ deletion construct indicates that the intermolecular hydrogen bond with D266 is indeed favorable for c-di-GMP binding (Fig. 5*B*) in the PilF background. This is also supported by additional ITC data for PilF_159-302_ binding to cyclic dimeric inosine-monophosphate (c-di-IMP, Fig. 5*C*), which lacks the amino groups at position 2 in the guanine bases and binds PilF_159-302_ with a K_D_ value of 60 ± 1 nM that is very similar to the K_D_ for PilF_159-221_ and c-di-GMP (Fig. 5*D*).

In summary, we find that in the context of PilF GSPII-B, the C-terminal subdomain contributes favorably to c-di-GMP binding by PilF_159–302_, which is in contrast to MshEN and further rationalizes the extremely high affinity of PilF_159-302_ for c-di-GMP.

### c-di-GMP binding requires the first turn of helix α1 in PilF_159-302_ to unfold

To investigate possible conformational rearrangements of PilF159-302 upon c-di-GMP recognition in order to characterize the ligand-binding mode in more detail, we solved the NMR solution-structure of PilF_159-302_ in the apo state.

**Figure 6:**
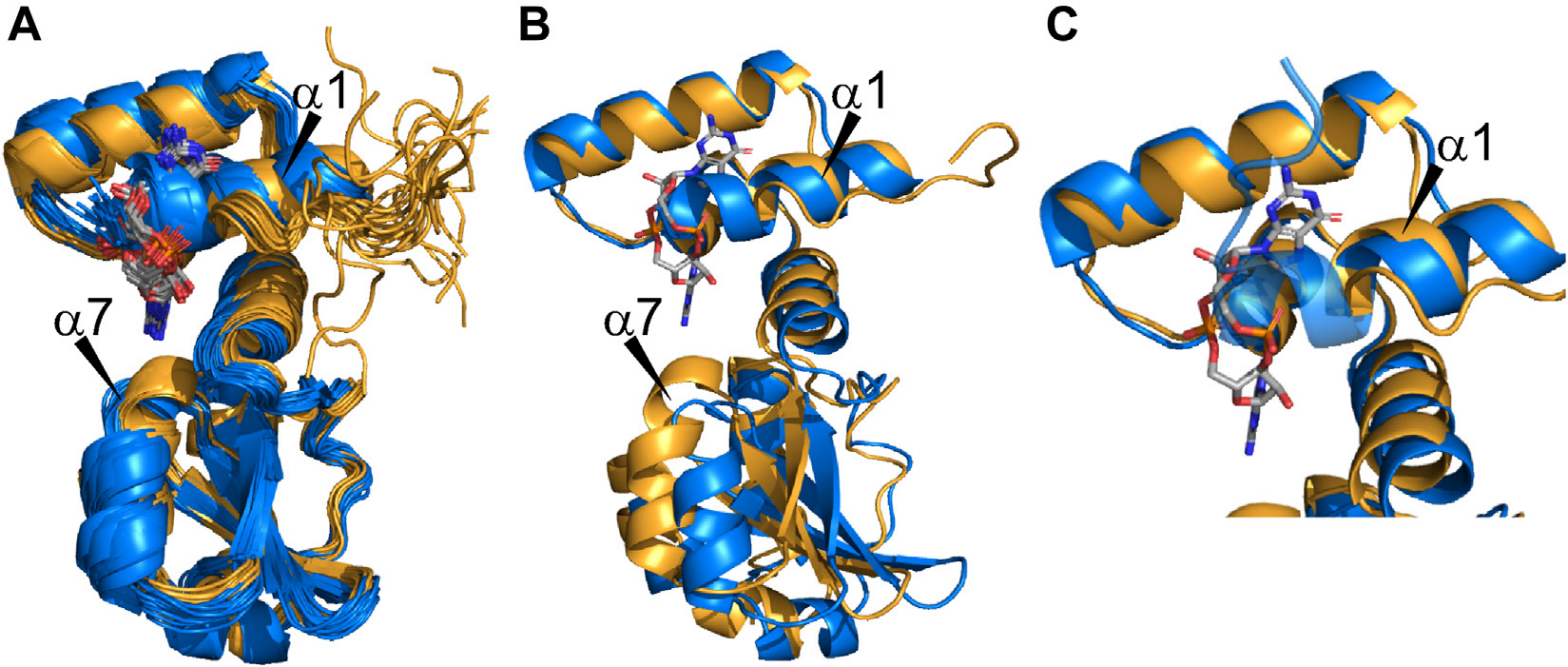
**The apo state of PilF**_**159–302**_**has an elongated first α-helix.***A*, the NMR-solution structure of apo PilF_159–302_ (*blue*) is presented as a bundle of the 20 lowest energy CYANA structures. It exhibits a fold nearly identical compared to the c-di-GMP–bound state of PilF_159–302_ (*yellow*). *B*, superimposition of the N-terminal subdomains of PilF_159-302_ in the apo (*blue*)- and the holo (*yellow*)-state. The two structures with the lowest target function were used. *C*, close-up of the superimposed N-terminal subdomains. The helix α1 is N-terminally elongated by one turn in the apo-state (*blue*) and reaches into the c-di-GMP–binding site.

Based on thorough backbone and sidechain assignment presented previously (30), we calculated the 20 lowest energy structures of apo-PilF_159-302_ deduced from 2754 NOE-distance restraints and 232 dihedral angle restraints. The resulting structural bundle is well defined with backbone and heavy atom RMSD values of 0.45 and 0.89 Å, respectively, a target function as low as 1.09 without distance restraint or torsion angle restraint or Ramachandran violations (Fig. 6*A*). The apo and the holo state of PilF_159-302_ both act as uniformly rigid units with no extended flexible regions apart from the N- and C-termini as shown by {^1^H},^15^N-hetNOE and ^15^N-spin relaxation data (Fig. S15).

The global fold of apo-PilF_159-302_ is very similar to holo-PilF_159-302_ and rigid parts of both structures align with an overall RMSD of 1.3 Å (corresponding to residues 167–300). Interestingly, it becomes evident that the first α-helix is N-terminally elongated by one turn in the apo state of PilF_159-302_, which is confirmed by the α-helix typical NOE-pattern (Figs. 6*A* and S16). As a consequence of the presence of the additional turn of helix α1 in the apo state, the side chain of K164 is oriented towards the side chain of L196 in α4 creating an additional hydrophobic packing interaction that is not present when the ligand is bound and this helical turn is unfolded (Fig. S17). A superposition of apo- and holo-PilF_159-302_ with the N-terminal subdomains (RMSD 1.5 Å) shows that the subdomain structures are nearly identical apart from helix α1. However, a small difference is obvious in the relative orientation of the two subdomains with respect to each other. In the c-di-GMP–bound state, the C-terminal subdomain is tipped towards the N-terminal subdomain and helix α7 is shifted upwards towards the c-di-GMP–binding site highlighting its involvement in c-di-GMP binding (Fig. 6*B*). Furthermore, the superposition of the C-terminal subdomains results in an RMSD value of 1.1 Å. The superposition highlights that in the c-di-GMP–bound state helix, α7 is N-terminally elongated (Fig. S18). Both the change in relative orientation of the subdomains and the elongated helix α7 compared to the apo state of PilF_159-302_ are directly related to the interaction of D266 and R268 with the amino group of c-di-GMP described above. The superposition of the N-terminal subdomains in apo-PilF_159-302_ and holo-PilF_159-302_ clearly show that the elongated state of helix α1 in the apo state reaches into the c-di-GMP–binding site leading to steric clashes with c-di-GMP (Fig. 6*C*). For c-di-GMP to fit into the PilF_159-302_–binding pocket, the first turn of helix α1 must unfold to enable the protein–ligand interactions described in the preceding sections with minimal additional structural rearrangements. To test this point, we created the mutant PilF_159-302_ L166G to interrupt the first helical turn by the introduction of a glycine residue. The CSI derived from the backbone NMR resonance assignment of this mutant showed clearly that now in the apo protein helix, α1 is one turn shorter than in the WT (Fig. S19). ITC measurements showed that the affinity of the mutant for c-di-GMP was increased yielding a reduced K_D_ value of 3 nM (Fig. S20). Both triangular leucine clusters on which the c-di-GMP base surfaces stack are pre-formed in the apo-state and L196 is in perfect position to stack on the surface of one guanine base facing away from the protein core (Fig. S21, *A* and *B*). Moreover, Q190 is in perfect position to enable straightforward hydrogen bond formation to the ribose-phosphate backbone of the ligand (Fig. S21*C*). Only K167 and Q218 are oriented differently compared to holo-PilF_159-302_. For K167, this is due to the elongated helix α1. Q218 seems to be flexible in the apo-state seems with no defined conformation (Fig. S21*D*).

### Impairing c-di-GMP binding in PilF_159-302_ leads to a decreased twitching motility and adhesion capability of *T. thermophilus* cells

**Figure 7:**
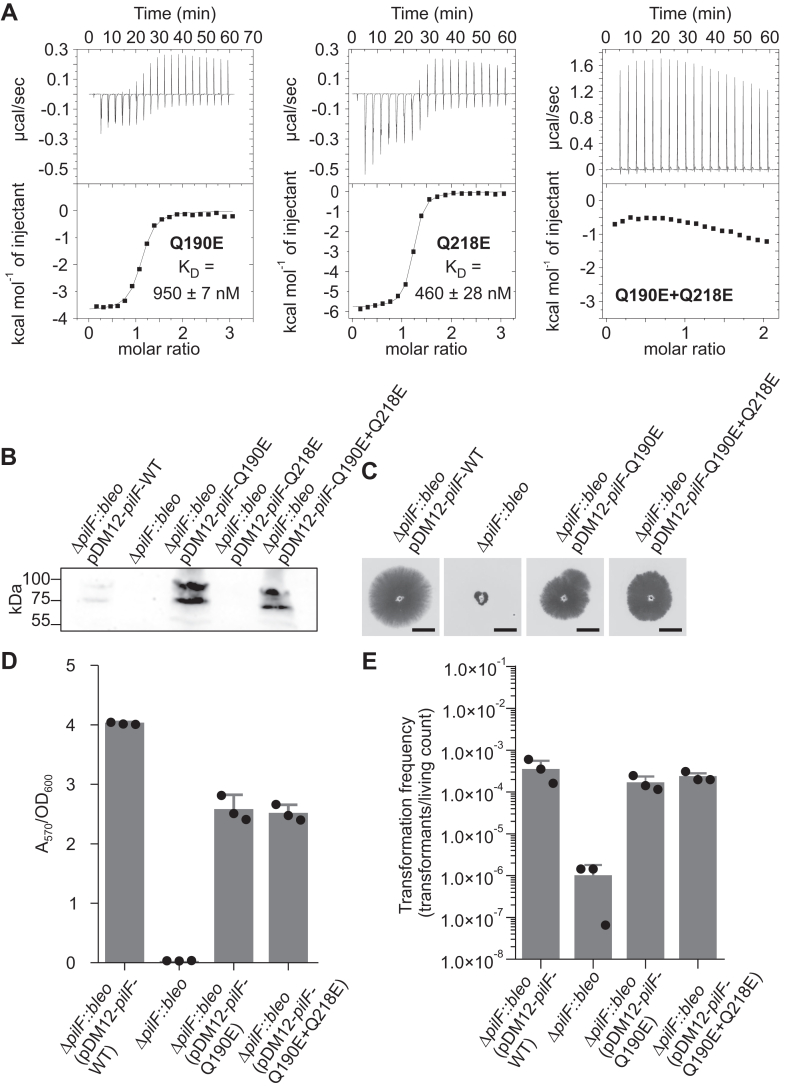
**Effects of c-di-GMP–binding impairment in PilF**_**159-302**_**on *T. thermophilus* physiology.***A*, c-di-GMP–binding assays of PilF_159-302_ variants Q190E (*left*), Q218E (*middle*), and Q190E + Q218E (*right*) using ITC. *B*, Western blot to confirm protein production of PilF variants in *T. thermophilus*. *C*, twitching motility assay of *T. thermophilus* cells possessing WT-PilF or the c-di-GMP–binding impaired PilF variants. *D*, cell adhesion assay of *T. thermophilus* with the PilF variants compared to WT-PilF. *E*, transformation frequency of *T. thermophilus* cells carrying WT-PilF compared to cells carrying the c-di-GMP–binding impaired PilF variants.

To evaluate the impact of c-di-GMP binding by PilF_159-302_ on PilF function in natural transformation and twitching motility, we created PilF constructs that are impaired in their c-di-GMP–binding capabilities based on our structural data. To ensure the structural integrity of the resulting PilF constructs, we aimed for minimal deviation from the WT-PilF sequence. By inspecting the c-di-GMP–binding pocket, we identified residues Q190 and Q218 as promising targets for mutations abolishing ligand binding for the PilF GSPII-B domain (see Fig. 3*C*). Due to the involvement of the Q190 and Q218 side chains in hydrogen bonds with the negatively charged ribose-phosphate backbone of c-di-GMP, we introduced glutamate residues at both positions as single and as double mutants (Q190E, Q218E, Q190E+Q218E). Thus, an oxygen atom preventing the formation of the hydrogen bond to the phosphate oxygen replaces the sidechain amino groups of the respective glutamines. Simultaneously, a negatively charged amino acid side chain is now in close proximity to the respective negatively charged phosphate groups of c-di-GMP. This in turn should further interfere with c-di-GMP binding due to unfavorable electrostatic interactions. The resulting PilF_159-302_ constructs Q190E, Q218E, and Q190E+Q218E were tested *in vitro* using ITC measurements to assess their c-di-GMP–binding capabilities (Fig. 7*A*). When Q190E and Q218E are tested individually in PilF_159-302_, both constructs show drastically lower affinities to c-di-GMP with PilF_159-302_ Q190E exhibiting a K_D_ value of 950 ± 7 nM and PilF_159-302_ Q218E a K_D_ value of 460 ± 28 nM. Compared to the WT-PilF_159-302_ affinity (6 nM), the Q190E construct has a 158 fold and the Q218E construct a 77-fold lower affinity to c-di-GMP but both are still very similar to the MshEN K_D_ value (500 nM) and are therefore potentially able to bind the ligand at biologically relevant concentrations. Thus, we introduced both mutations simultaneously into PilF, which successfully abrogated c-di-GMP binding in the resulting PilF_159-302_ Q190E+Q218E construct (Fig. 7*A*). Importantly, the structural integrity of this construct is not affected by the double mutation as obvious from a comparison of 1D-^1^H proton spectra of WT and mutant protein in their apo states (Fig. S22).

To test the biological consequences of disrupting c-di-GMP binding to the PilF GSPII-B domain, we introduced Q190E and Q218E mutations into full-length PilF. The respective constructs with either single or double point mutations were expressed in the ΔPilF::bleo strain of *T. thermophilus*, in which the genomic *pilF* gene is knocked out. Plasmid DNA encoding for the respective tested PilF constructs was used to complement the missing genomically encoded PilF and a Western Blot verified the presence of PilF *in vivo* (Fig. 7*B*). Unfortunately, PilF Q218E expression was not detectable *in vivo* (Fig. 7*B*). For PilF, Q190E and Q190E+Q218E expression could be verified and the cells were used for subsequent assays (Fig. 7, *C*–*E*). The single and double point mutations Q190E and Q190E+Q218E in PilF led to a ∼40% decrease in twitching motility and in cell adhesion indicating an effect of c-di-GMP binding to the PilF GSPII-B domain on pili formation or movement (Fig. 7, *C* and *D*). In contrast, the transformation efficiency of the mutants was similar to WT PilF, indicating that the ability of PilF GSPII-B to bind c-di-GMP is not important for the function of PilF in DNA uptake (Fig. 7*E*). In sum, we were able to impair c-di-GMP binding in PilF GSPII-B with minimal mutations and showed that the ability of PilF GSPII-B to bind c-di-GMP with high affinity is important for twitching motility and cell adhesion but has no influence on transformation efficiency. The molecular mechanism on how the information of binding the second messenger c-di-GMP to the PilF-GSPII domains is transferred to the *T. thermophilus* T4P that mediates twitching motility exceeds the scope of this work and remains the subject of future studies.

## Discussion

Here we present the structure of the second GSPII domain (GSPII-B) from the N-terminal part of the traffic ATPase PilF from *T. thermophilus* in complex with its ligand c-di-GMP and in its apo state. Based on structural data gained from NMR solution and X-ray structures, we are able to define conformational changes that occur upon c-di-GMP binding. Ligand binding in the N-terminal subdomain of the PilF GSPII-B domain causes only limited structural changes including unwinding of the N-terminal turn of helix α1 in the ligand-binding site as well as the reorientation of the C-terminal subdomain with respect to the N-terminal subdomain. While ligand recognition in the PilF GSPII-B domain is highly similar to the MshEN domain from *V. cholerae*, its c-di-GMP affinity is ∼ 83-fold higher (17). Based on structural comparisons and mutational studies, we found that the replacement of two critical N-terminal arginine residues in the ligand-binding motifs of MshEN by a lysine and a leucine residue, respectively, and the concomitant replacement of a cation-π-stacking interaction by an extended hydrophobic stacking interaction is an important contribution to the higher ligand affinity in the PilF GSPII-B domain. As a consequence, the proposed c-di-GMP–binding consensus sequence motif based on the MshEN structure should be extended from RLGxx(L/V/I) (L/V/I)xxG(L/V/I) (L/V/I)xxxxLxxxxLxxQ (17) to (R/K/L)LGxx(L/V/I) (L/V/I)xxG(L/V/I) (L/V/I)xxxxLxxxxLxxQ. Furthermore, our results in conjunction with previous affinity measurements for similar domains from other organisms suggest that the replacement of the N-terminal arginine of the motif by a more hydrophobic residue can serve as a predictor for very high affinity c-di-GMP binding. Notably, the hydrophobic amino acid replacements at the N-termini of the c-di-GMP–binding sequence motifs also render the PilF GSPII-B domain the first c-di-GMP–binding domain that recognizes its ligand without the involvement of an arginine residue. Another important difference in c-di-GMP binding between MshEN and the GSPII-B domain is the influence of the C-terminal subdomain on the ligand affinity. While both protein structures show a single hydrogen bond contact between an aspartate side chain from the C-terminal subdomain and an amino group of a guanine base, this contact is slightly detrimental for ligand affinity in MshEN most likely due to unfavorable entropic contributions (17). From our data, this contact is clearly advantageous for ligand affinity in the PilF GSPII-B domain as the side chain orientation of the aspartate is preorganized due to the presence of a salt bridge with an arginine side chain (Arg268).

The very high affinity of c-di-GMP binding to the PilF GSPII-B domain is of functional importance. A single point mutant (Q190E) shifts the K_D_ of this domain into the realm of the affinity observed for the original MshEN domain (∼500 nM) and likewise reduces twitching motility and surface adhesion of *T. thermophilus in vivo*. Notably, a full knock-out of c-di-GMP binding to this domain by a double mutant (Q190E+QE218E) does not lead to a more severe phenotype suggesting that it is indeed the high affinity and not the c-di-GMP–binding ability *per se* that impairs the function of PilF in twitching motility and surface adhesion. Remarkably, the transformability of *T. thermophilus* was not significantly impaired by a reduction or the complete loss of the c-di-GMP–binding ability of the GSPII-B domain of PilF. This indicates the functional specialization of the three GSPII domains in PilF. In conjunction with previous domain deletion experiments which showed that the entire GSPII-A domain can be deleted without influencing transformation efficiency (7), this suggests that GSPII-C and/or its c-di-GMP–binding activity might be involved in the regulation of the DNA uptake by PilF. This is also consistent with the previous finding that deletion of the GSPII-C domain leads to hypertransformability (7).

Intracellular c-di-GMP concentrations in bacteria are generally found to be in a range between ∼50 nM and ∼50 μM (33). The concentration fluctuates with growth phase, environmental conditions, and changes in life style with particularly low concentrations measured in the early growth phase. Natural transformation frequencies and pilus biogenesis are known to be modulated in the dependence of environmental conditions and growth phase. In the Gram-negative pathogen *Acinetobacter baumannii*, the highest transformation frequencies were detected in a tight window starting 90 min after inoculation (34) whereas in *Acinetobacter baylyi*, maximal transformation frequencies were detected immediately after inoculation into fresh medium and decreased during prolonged exponential growth (35). The production of pili and the T4P-mediated twitching motility has been found to be growth phase and cell density dependent in different bacteria, such as *A. baumanii* (34), *Legionella pneumophila* (36), and *Ralstonia solanacearum* (37). The question whether DNA uptake and T4P biogenesis depend on the growth phase in *T. thermophilus* cannot be clearly answered yet, but previous studies suggest highest transformation frequencies directly after inoculation into fresh medium (38). Environmental factors such as temperature or medium composition have been shown to impact piliation of *T. thermophilus*. At a lower growth temperature of 55 °C, cells displayed a hyper-piliation and increased adhesion to solid surfaces whereas transformation frequencies remained unchanged (39). However, for some species such as *E. coli*, it was shown that global cellular c-di-GMP concentrations stay as low as ∼ 100 nM throughout its growth cycle (40) but reach higher local concentrations. The K_D_ of the PilF GSPII-B domain for c-di-GMP binding is significantly lower than these concentrations raising questions with regard to the biological importance of the ligand-free state of this domain since it should be predominantly in the c-di-GMP–bound state under most conditions. Thus, it might be possible that c-di-GMP binding to GSPII-B is only important in very early growth phases where c-di-GMP concentrations are particularly low. Alternatively, c-di-GMP might not be required to regulate the function of this domain but rather required as a structural component in forming a composite surface potentially involved in PilF binding to other proteins. On the other hand, the effective concentration of free c-di-GMP might be lower than the total measured concentration since a large fraction of c-di-GMP might be bound by other effectors that might be more abundant than PilF or have higher or similar c-di-GMP affinities as has been described in particular for RNA receptors for c-di-GMP (41, 42, 43). In this regard, it should be noted that actual estimates for the total number of c-di-GMP molecules in bacterial cells is in the range of a few tens to a few hundred molecules (44).

The second messenger c-di-GMP has to be sensed and there is a great diversity of c-di-GMP effectors or targets. c-di-GMP can interact with RNAs, that is, riboswitches, or diverse classes of proteins. However, nothing is known with respect to c-di-GMP effectors in *T. thermophilus* so far. c-di-GMP is synthesized from GTP by diguanylate cyclases which contain a highly conserved active site (GGDEF domain). Many also contain a secondary c-di-GMP–binding site (I-site) (45, 46). Degradation of c-di-GMP is mediated by specific phosphodiesterases (PDEs) which are characterized by EAL or HD-GYP domains. Many diguanylate cyclases and PDEs contain both, GGDEF and EAL domains, where one domain is enzymatically inactive and exerts a regulatory role. The genome of *T. thermophilus* HB27 encodes multiple potential c-di-GMP–binding effectors, such as nine potential GGDEF or/and EAL domain proteins. These proteins are good candidates to function as c-di-GMP–sensing effectors such as I) controlling the activity of a target (protein or promoter) or II) binding and degradation of c-di-GMP. That enzymatically active EAL domain proteins indeed function as c-di-GMP effectors has been shown recently, such as for the EAL domain PDE PdeR of *E. coli* (47).

One potential consequence of c-di-GMP binding to PilF GSPII-domains could be a modulation of potential interdomain interactions. However, the similarity of the measured dissociation constants between the different constructs ranging from the hexameric full-length protein to the isolated GSPII-B domain argue against extensive interdomain interactions and their allosteric modulation by c-di-GMP binding. However, a more detailed investigation of potential interdomain interactions will be subject to future experiments.

## Experimental procedures

### Plasmids and construct design

To introduce mutations in the key positions of PilF_159-302_ and to create truncated constructs, a pET-11a vector containing the WT PilF_159-302_ coding sequence with a preceding TEV-cleavage site and a hexahistidine tag (His-6) was used (His-6-TEV-PilF_159-302_-pET-11a). A two-step site-directed mutagenesis protocol was applied to create point mutation constructs (48).

The C-terminally truncated construct PilF_159-221_ was created by standard Gibson assembly (49) using the oligonucleotide pair PilF-159/PilF-221 and the His-6-TEV-PilF_159-302_-pET-11a as a template.

To complement the *T. thermophilus ΔpilF::bleo* mutant (50) with the *pilF*_Q190E_, *pilF*_Q218E_, and *pilF*_Q190E + Q218E_ genes, the point mutations were introduced in *pilF* in the plasmid pET28a-*pilF*-WT by standard targeted mutagenesis (51). The phosphorylated primer pairs Q190E_for and Q190E_rev as well as Q218E_for and Q218E_rev were used to introduce the mutations (Table S5). The double point mutations were introduced in a two-step process using pET28a-*pilF*-Q218E as a template with the Q190E_for and Q190E_rev primers. The PCR-products were ligated and transformed into *E. coli* TOP10 (New England Biolabs). For selection, 20 μg/ml kanamycin were added. After successful generation of pET28a-*pilF*-Q190E, pET28a-*pilF*-Q218E and pET28a-*pilF*-Q190E+Q218E, the mutated genes we recloned into the pDM12 vector (52) using *NdeI* and *NotI* restriction sites. Selection was performed with 20 μg/ml kanamycin and 100 μg/ml ampicillin. The resulting plasmids pDM12-*pilF*-Q190E, pDM12-*pilF*-Q218E, and pDM12-*pilF*-Q190E+Q218E were introduced to the *T. thermophilus ΔpilF::bleo* mutant by electroporation as described previously (50). *T. thermophilus* was grown in TM^+^-medium (8 g/l tryptone, 4 g/l yeast extract, 3 g/l NaCl, 0.6 mM MgCl_2_, and 0.17 mM CaCl_2_) at 68 °C. If needed, kanamycin (20 μg/ml), bleomycin (35 μg/ml), or streptomycin (100 μg/ml) were added.

### Protein expression and purification

For protein production, commercially obtained pET-11a plasmids (GenScript) encoding the protein-constructs PilF_1-889_, PilF_1-482_, PilF_159-482_, PilF_1-302_, and PilF_159–302_, respectively, were used as described previously (30). All constructs contained N-terminal hexahistidine tags followed by TEV-cleavage sites. All proteins were heterologously expressed in the *E. coli* BL21 [DE3] *Gold* cell line (Agilent Technologies/Stratagen). TEV-cleavage of the His_6_-tag resulted in proteins with either 889 (PilF_1-889_), 482 (PilF_1-482_), 323 (PilF_159-482_), 302 (PilF_1-302_), 144 (PilF_159–302_), or 63 (PilF_159–221_) native residues with two additional artificial residues (Gly and Ser) at the N-terminus. The numbering scheme refers to the full-length PilF of *T. thermophilus*. Protein expression and purification of unlabeled and uniformly ^15^N or ^15^N,^13^C labeled PilF_159–302_, PilF_159–221_ and their point mutants was performed as described before (30). All other proteins were purified by a combination of Ni-NTA affinity and size-exclusion chromatography. Expression of selectively and stereo-specifically–labeled protein constructs essentially followed the previously described protocols. To isotopically label specific amino acids, the respective precursor was added to the M9-medium 1 h prior to the induction of protein production *via* the addition of 1 mM IPTG. For selective ^13^C-labeling of leucine and valine methyl groups, 120 mg/l of the precursor Keto-3-(methyl-^13^C)-butyric-4-^13^C acid sodium salt (SigmaAldrich) was added to the expression medium (53). To label leucine and valine methyl groups stereo-specifically, a mixture of 10% ^13^C-glucose and 90% unlabeled glucose was introduced as the carbon source (54). All steps before and after the addition of the respective precursor or the usage of a glucose mixture are identical to the protocol described before (30).

For the investigation of c-di-GMP binding by PilF_159–302_ *via* NMR-spectroscopy, unlabeled and ^13^C,^15^N labeled c-di-GMP was synthesized using a constitutively active mutant of a diguanylate cyclase from the thermophilic bacterium *Thermogata maritima.* ^13^C,^15^N labeled GTP was used as a substrate to produce labeled c-di-GMP (55, 56).

### Isothermal titration calorimetry

To assess the ligand-binding capabilities of the various PilF constructs, triplicates of ITC measurements were recorded for each construct. The measurements were carried out using a MicroCal ITC_200_ (Malvern Panalytical). All ITC experiments were conducted at 20 °C with 50 mM Tris-HCl pH 7.5, 200 mM sodium chloride, 1 mM β-mercaptoethanol as the sample buffer. All protein samples were extensively dialyzed against the sample buffer and the buffer used for dialysis was used to dilute protein and c-di-GMP samples in preparation for the ITC measurements. Each measurement was conducted using the following protocol. In total, 39.8 μl c-di-GMP were titrated to 270 μl protein in 20 separate injections in 180 s intervals with a stirring speed of 750 rpm. After an initial delay of 120 s, the first injection of 0.2 μl c-di-GMP with a duration of 0.4 s was followed by 19 injections of 2 μl with a duration of 4 s. During the measurement, the feedback mode was set to high and the reference power to 11 μcal s^−1^. To optimize precision of the measurements, protein and c-di-GMP concentrations were adjusted to the binding capabilities of the respective constructs and ranged from 5 to 200 μM for protein and 50 to 2000 μM for c-di-GMP. All thermograms were processed using Origin 7.0 (OriginLab) using a one-site– or two-site–binding model dependent on the number of expected binding events.

### NMR spectroscopy

NMR-samples for assignment, structure, and dynamics determination were prepared in 50 mM Bis-Tris pH 5.8, 200 mM NaCl, 1 mM β-mercaptoethanol, and 6.75% (v/v) D_2_O. Isotopically labeled proteins were used at a concentration of 500 to 540 μM; isotopically labeled c-di-GMP was used at a concentration of 420 μM in separate samples. NMR-titration experiments were carried out in 200 μM samples of PilF_159-302_ in the same buffer. All NMR-spectra were recorded at 45 °C on Bruker AVANCE 600, 700, 800, 900, and 950 MHz spectrometers equipped with cryogenic triple resonance probes and processed using Bruker TOPSPIN versions 3.5 and 4.0.7. Proton resonances were internally referenced to 200 μM 2,2-dimethyl-2-silapentane-5-sulforic acid and appropriate conversion factors were applied to indirectly reference ^13^C and ^15^N chemical shifts (57). Resonance assignments of bound c-di-GMP and PilF_159-302_ in the apo as well as the c-di-GMP–bound state of PilF_159-302_ were carried out in CARA (58) based on a set of triple resonance experiments as described earlier (30). Stereospecific assignments of the leucine/valine methyl groups were analyzed using CARA as well as CcpNmr Analysis (59). The assignment is based on the presence (pro-R) and the absence (pro-S) of a scalar coupling between the methyl-group carbon atom and the covalently bound carbon atom (Cβ in valines and Cγ in leucines), respectively. Accordingly, that leads to the presence or the absence of a resonance splitting of the respective methyl groups in the ^13^C dimension in a stereospecifically labeled sample.

To assess fast time scale dynamics of the apo and the holo state, {^1^H},^15^N-heteronuclear Overhauser effect (HetNOE) experiments were recorded using standard Bruker pulse sequences with 500 μM PilF_159–302_ in the apo and with 541 μM PilF_159–302_ in the c-di-GMP–bound state (812 μM c-di-GMP). For each state, two hetNOE experiments were conducted at 600 MHz in succession with and without proton saturation during the recovery delay (40). Peak volumes of each experiments were extracted by integration in TopSpin 4.0.7 and the values of each set of experiments were averaged. Final hetNOE values (ΔI) were calculated using (Equation 1):

(1) $$\Delta I=\frac{I_{x}}{I_{0}}$$

where I_x_ is the peak integral with and I_0_ without proton saturation.

^15^ N longitudinal (R_1_) and transversal (R_2_) spin relaxation experiments were recorded at a Bruker AVANCE 600 MHz spectrometer in an interleaved fashion using standard Bruker pulse sequences and uniformly ^15^N labeled samples. Concentrations of 500 μM PilF_159–302_ in the apo state and 484 μM PilF_159–302_ in the c-di-GMP–bound state (727 μM c-di-GMP) were used. Relaxation decay times used for determining R_1_ and R_2_ relaxation rates were set to 800, 50, 300, 100, 1300, 500, 200, 400, 1000, 600, 10, and 1600 ms and 17, 34, 68, 136, 17, 204, 238, 51, 272, 102, 34, and 85 ms in this order, respectively. Peak volumes (I) were assessed in TopSpin 4.0.7 and fitted in R version 4.0.2 with (Equation 2):

(2) $$I=a*e^{t*R_{x}}$$

where *a* is the peak volume at 0 ms decay time, *t* is the relaxation decay time, and *R*_*x*_ is either R_1_ or R_2_.

To assess the ribose sugar pucker of bound c-di-GMP, a forward-directed HCCH-TOCSY-CCH-E.COSY (60) was recorded. The sample contained unlabeled PilF_159-302_ (367 μM) bound to ^13^C,^15^N-labeled c-di-GMP (440 μM).

### Direct detection of intermolecular hydrogen bonds by NMR

The presence of hydrogen bonds between amide protons of the protein backbone and the phosphate groups of c-di-GMP was directly detected by recording a 2D-^1^H,^31^P-SOFAST-HMQC spectrum (61, 62) optimized for magnetization transfer across the hydrogen bond from the amide proton (H^N^) to the phosphate group of c-di-GMP. The spectrum was recorded at 45 °C using a sample of 420 μM unlabeled PilF_159–302_ and 420 μM unlabeled c-di-GMP. To detect H^N^-N hydrogen bonds, a 2D-HNN-COSY spectrum (63) optimized for RNA was recorded using a sample containing 940 μM ^15^N-labeled PilF_159–302_ and 1122 μM ^13^C,^15^N-labeled c-di-GMP.

### Structural restraints

To assess the tertiary structure of PilF_159-302_ in the apo- and the holo-state, distance restraints based on the NOE-data were deduced from ^15^N- and ^13^C-NOESY-HSQC spectra in H_2_O with mixing times of 120 ms using uniformly ^15^N and ^13^C,^15^N-labeled samples, respectively. For aliphatic and aromatic carbons, the spectra were optimized for the respective carbon resonance frequencies and the corresponding ^1^J_CH_ coupling constants. Due to the abundance of leucine residues, additional ^13^C-NOESY-HSQC spectra optimized for methyl group resonance frequencies were recorded in H_2_O with a mixing time of 120 ms to enhance resolution and to deduce specific distance restraints involving the methyl groups of leucines and valines using the methyl group–labeled samples. Additionally, torsion angle restraints were calculated with TALOS-N (64) based on H^N^, N, Cα, Cβ, and CO chemical shifts.

All peaks were picked manually and evaluated in CcpNmr-Analysis (59). Subsequent NOE assignments and structure calculations were performed in an automated fashion with CYANA (65). All resulting peak lists including NOE assignments were manually reviewed in CcpNmr-Analysis (59) and corrected in the case of artifacts.

The assignment of ∼90% of all observable cross-peaks was achieved for all NOESY spectra of the PilF_159-302_ apo state.

For PilF_159–302_ bound to c-di-GMP, additional intermolecular NOEs were identified in 2D-^13^C-NOESY-HSQC and 2D-^15^N-amino-NOESY-HSQC spectra optimized for RNA using a sample with 420 μM unlabeled PilF_159–302_ and 420 μM ^13^C,^15^N labeled c-di-GMP. These NOEs were included in structure calculations and evaluated as described above. In sum, all observable intermolecular NOE cross-peaks were assigned. Directly detected intermolecular hydrogen bonds between the protein backbone and c-di-GMP were included as lower and upper limit distance restraints. In the same manner, two additional hydrogen bonds between the protein backbone and c-di-GMP were included in the structure calculation. Both hydrogen bonds derive from chemical shift perturbation data and D_2_O exchange experiments.

### Structure calculations

Structure calculations of both the PilF_159-302_ apo- and holo-state followed the same cycle of automated NOE-assignment, distance restraint extraction, structure calculation, evaluation, and corrective input until target function and RMSD value are at a minimum, and Ramachandran plot outliers, distance, van der Waals, and torsion angle violations are eliminated. In each cycle, 100 conformers were calculated by CYANA using 20.000 torsion angle molecular dynamics steps and the 20 structures with the lowest target function were selected to represent the respective solution structures. The 20 best structures were submitted to the restrained energy refinement program OPALp, which subjects the structure to the AMBER94 force field for energy minimization (66).

For the c-di-GMP–bound state of PilF_159-302_, an artificial linker of pseudo-atoms was introduced in the protein sequence and the CYANA library file to link PilF_159-302_ to the c-di-GMP molecule during the structure calculations (67). The structure of the c-di-GMP–bound state of PilF_159-302_ was calculated as described above. The NMR statistics for the structures of PilF_159-302_ in the apo and the c-di-GMP–bound state are listed in Table 1.

### Crystallization of the PilF_159–302_ c-di-GMP complex

Prior to crystallization, protein and c-di-GMP were mixed in a molar ratio of 1:1.5 and incubated for 20 min at room temperature. Crystallization of native PilF_159–302_ in complex with c-di-GMP followed the hanging-drop-vapor-diffusion protocol at 291 K in 24 well crystallization trays with 1.8 M LiSO_4_, 0.1 M sodium acetate pH 5.5 as a precipitant. Crystallization of PilF_159-302_ constructs K167L and K167R followed the same protocol except for slightly adjusted precipitant concentrations. For the K167R and K167L constructs, LiSO_4_ concentrations were decreased to 1.5 M while sodium acetate concentrations and pH value were kept constant. For all constructs, the precipitant solution (1 μl) was mixed with the protein solution (1 μl, 15 mg ml^–1^) and diamond-shaped crystals were collected after 1 week. Before flash-freezing in liquid nitrogen, the protein crystals were thoroughly washed in 0.5 M LiSO_4_, 0.1 M Na-acetate pH 5.5, and 33% (v/v) PEG 400 for cryoprotection.

### Diffraction data collection and refinement

Crystals of native PilF_159-302_ bound to c-di-GMP were tested for diffraction at the Paul Scherrer Institut at SLS, Villigen, Switzerland at the NSLS Beamline X6A with a diffraction up to 2.0 Å. Data were collected at a wavelength of 1.0 Å at 100 K. Indexing and integration were carried out with the XDS processing software (68) and the datasets were merged with Pointless and Aimless (69, 70, 71) resulting in a dataset with an overall completeness of 92.9% and an R_merge_ of 4%. Coordinates of the solution NMR-structure of the PilF_159–302_-c-di-GMP complex presented here were used as a starting point for molecular replacement in PHENIX (72). Iterative model rebuilding, refinement, and quality assessment was performed with PHENIX and WinCoot (72, 73, 74).

Crystals for PilF_159-302_ mutants K167L and K167R in complex with c-di-GMP were tested for diffraction at the P13 beamline of Deutsches Elektronen-Synchrotron (DESY), Hamburg, Germany. The K167L mutant diffracted up to 1.9 Å and the K167R mutant diffracted up to 1.8 Å. For both mutants, data were collected at 100 K at a wavelength of 0.976 Å. Data processing and structure refinement were conducted as described above. For the K167L mutant, the dataset had a completeness of 100% with an R_merge_ value of 7% and the K167R dataset had a completeness of 100% with an R_merge_ of 7%. Detailed data collection and refinement statistics are presented in Table 2.

In the structural analysis, hydrogen bonds were considered to be present when the distance between the heavy atoms of the hydrogen bond donor and acceptor groups was < 3.2 A and when the hydrogen bond angle was >145°.

### Western blot analysis

Western blot analyses of PilF and all variants was performed as previously described (11) using a polyclonal PilF-antiserum at a dilution of 1:7500.

### Twitching motility studies

Twitching motility was analyzed on minimal medium agar plates containing 0.1% BSA as described previously (50). Mutant cells were applied by stab-inoculation and the plates were incubated at 68 °C for 3 days in a humid environment. The agar surface was subsequently stained with Coomassie Brilliant Blue, cells were removed, and the clear twitching zones were documented. To enhance contrast, the images were digitally inverted. Representative data from three independent experiments are shown.

### Adhesion studies

Adhesion to plastic surfaces was studied using microtiter plates as described previously (12). The cells were incubated in TM^+^ at 68 °C for 3 days in a humid environment. Subsequently, the final OD_600_ of the cultures was measured in triplicate. The medium was removed and the remaining cells were stained with 0.1% crystal violet. The crystal violet was dissolved in alcohol and the absorption was measured at 570 nm in triplicate. The adherence coefficient is given as ratio of A_570_/OD_600_. Representative data from three independent experiments are shown.

### Transformation studies

Natural transformation was studied on TM^+^ medium plates containing 2% agar using 5 μg of genomic DNA of a spontaneously streptomycin-resistant HB27 mutant as described previously (75). The frequency of transformation is given as the number of transformants per living count. Representative data from three independent experiments are shown.

## Data availability

All presented structures are deposited in the PDB. The X-ray and NMR structures of native PilF_159-302_ in complex with c-di-GMP were deposited with the accession numbers 8PDK and 8PKZ, respectively. The NMR-structure of PilF_159-302_ without c-di-GMP has the accession number 8PQU. The X-ray structures of the K167R and K167L constructs of PilF_159-302_ in complex with c-di-GMP were deposited with the accession numbers 8PFA and 8PE0, respectively.

## Supporting information

This article contains supporting information.

## Conflict of interest

The authors declare that they have no conflicts of interest with the contents of this article.
