# Supplementary material for: The structural basis for high-affinity c-di-GMP binding to the GSPII-B domain of the traffic ATPase PilF from Thermus thermophilus
Source: J Biol Chem. 2024 Nov 29;301(1):108041. doi: 10.1016/j.jbc.2024.108041 (PMC11731258; doi:10.1016/j.jbc.2024.108041)
Supplement: Supplemental data [file mmc1.pdf]

Supporting information

The structural basis for high-affinity c-di-GMP binding to the GSPII-B domain of the traffic ATPase PilF from *Thermus thermophilus*

Konstantin Neißner[1,2], Heiko Keller[1,2], Lennart Kirchner[3], Stefanie Düsterhus[1], Elke Duchardt-Ferner[1,2], Beate Averhoff[3], Jens Wöhnert[1,2]

[1]Institute for Molecular Biosciences, Goethe-University Frankfurt/M., Max-von-Laue-Str. 9, 60438 Frankfurt, Germany

[2]Center for Biomolecular Magnetic Resonance (BMRZ), Goethe-University Frankfurt/M., Max-von-Laue-Str. 9, 60438 Frankfurt, Germany

[3]Molecular Microbiology and Bioenergetics, Institute for Molecular Biosciences, Goethe-University Frankfurt/M., Max-von-Laue-Str. 9, 60438 Frankfurt, Germany

Supplementary Table 1.

Overview of the thermodynamic parameters for all ITC titration experiments described in the course of this paper.

**Construct**	**Ligand**	**Degrees [°C]**	**N**	**K_D [nM]**	**ΔH [cal/mol]**	**ΔS [cal/mol/deg]**
**PilF1-889**	c-di-GMP	20	1 = 0.72 ± 0.10<br>2 = 0.59 ± 0.03	1 = 324 ± 2<br>2 = 10 ± 5	1 = -11763 ± 780<br>2 = -15683 ± 970	1 = -10.4 ± 2.7<br>2 = -16.5 ± 2.2
**PilF1-482**	c-di-GMP	20	1 = 0.98 ± 0.04<br>2 = 0.85 ± 0.03	1 = 461 ± 36<br>2 = 17 ± 1	1 = -12793 ± 95<br>2 = -17536 ± 128	1 = -14.6 ± 0.2<br>2 = -24.3 ± 0.3
**PilF159-482**	c-di-GMP	20	1 = 1 ± 0.1<br>2 = 0.7 ± 0.1	1 = 208 ± 27<br>2 = 1.0 ± 0.3	1 = -8689 ± 959<br>2 = -11766 ± 1182	1 = 0.95 ± 3.5<br>2 = 2.1 ± 4.7
**PilF1-302**	c-di-GMP	20	0.94 ± 0.13	7 ± 2	-14263 ± 2001	-11.4 ± 6.4
**PilF159-302**	c-di-GMP	20	0.66 ± 0.02	6 ± 1	-10795 ± 1145	-0.8 ± 4.0
**PilF159-302 K167R**	c-di-GMP	20	0.79 ± 0.03	150 ± 7	-10420 ± 552	-4.4 ± 1.9
**PilF159-302 K167L**	c-di-GMP	20	0.76 ± 0.10	2 ± 0.3	-10241 ± 494	5.4 ± 1.4
**PilF159-221**	c-di-GMP	20	1.17 ± 0.02	54 ± 3	-11453 ± 189	-5.8 ± 0.6
**PilF159-302 D266A**	c-di-GMP	20	0.95 ± 0.05	27 ± 2	-9995 ± 161	0.6 ± 0.4
**PilF159-302 Q190E**	c-di-GMP	20	1.11 ± 0.03	950 ± 7	-3389 ± 156	15.4 ± 0.4
**PilF159-302 Q218E**	c-di-GMP	20	1.26 ± 0.06	460 ± 28	-5312 ± 340	10.9 ± 1.0
**PilF159-302 L196R**	c-di-GMP	20	1.23 ± 0.03	23 ± 2	-8148 ± 209	7.1 ± 0.8
**PilF159-302 K167R+E170D**	c-di-GMP	20	0.76 ± 0.03	92 ± 8	-12843 ± 363	-11.6 ± 1.3
**PilF159-302 L166G**	c-di-GMP	20	0.92 ± 0.10	3 ± 0.5	-10283 ± 189	1.2 ± 3.4
**PilF159-302**	c-di-IMP	20	0.74 ± 0.03	60 ± 1	-7103 ± 234	8.8 ± 0.8
**PilF159-302**	c-di-GMP	45	0.88 ± 0.11	20 ± 2	-13327 ± 1389	-6.6 ± 4.2

Supplementary Table 2.

Overview of pairwise RMSD values for the backbone heavy atoms of all chains in the crystal structures of MshEN (5htl) and all $PilF_{159-302}$ variants.

RMSD	MshEN-A	MshEN-B	PilF159-302 WT-A	PilF159-302 WT-B	PilF159-302 K167R-A	PilF159-302 K167R-B	PilF159-302 K167L-A	PilF159-302 K167L-B
**MshEN-A**	0	0.902	1.449	1.486	1.394	1.490	1.465	1.502
**MshEN-B**	0.902	0	1.745	1.604	1.702	1.657	1.789	1.645
**PilF159-302 WT-A**	1.449	1.745	0	0.287	0.109	0.329	0.168	0.384
**PilF159-302 WT-B**	1.486	1.604	0.287	0	0.304	0.120	0.337	0.170
**PilF159-302 K167R-A**	1.394	1.702	0.109	0.304	0	0.299	0.164	0.374
**PilF159-302 K167R-B**	1.490	1.657	0.329	0.120	0.299	0	0.314	0.145
**PilF159-302 K167L-A**	1.465	1.789	0.168	0.337	0.164	0.314	0	0.295
**PilF159-302 K167L-B**	1.502	1.645	0.384	0.170	0.374	0.145	0.295	0

Supplementary Table 3.

Overview of pairwise RMSD values for the backbone heavy atoms of the N-terminal subdomains for all chains in the crystal structures of MshEN (5htl) and all $PilF_{159\text{-}302}$ variants.

**RMSD**	**MshEN-A**	**MshEN-B**	**PilF159-302 WT-A**	**PilF159-302 WT-B**	**PilF159-302 K167R-A**	**PilF159-302 K167R-B**	**PilF159-302 K167L-A**	**PilF159-302 K167L-B**
**MshEN-A**	0	0.212	0.806	0.838	0.711	0.847	0.789	0.770
**MshEN-B**	0.212	0	0.831	0.729	0.804	0.742	0.842	0.732
**PilF159-302 WT-A**	0.806	0.831	0	0.603	0.111	0.619	0.107	0.558
**PilF159-302 WT-B**	0.838	0.729	0.603	0	0.599	0.086	0.603	0.098
**PilF159-302 K167R-A**	0.711	0.804	0.111	0.599	0	0.612	0.154	0.558
**PilF159-302 K167R-B**	0.847	0.742	0.619	0.086	0.612	0	0.314	0.145
**PilF159-302 K167L-A**	0.789	0.842	0.107	0.603	0.154	0.314	0	0.551
**PilF159-302 K167L-B**	0.770	0.732	0.558	0.098	0.558	0.145	0.551	0

Supplementary Table 4.

Overview of pairwise RMSD values for the backbone heavy atoms of the C-terminal subdomains for all chains in the crystal structures of MshEN (5htl) and all $PilF_{159\text{-}302}$ variants.

**RMSD**	**MshEN-A**	**MshEN-B**	**PilF159-302 WT-A**	**PilF159-302 WT-B**	**PilF159-302 K167R-A**	**PilF159-302 K167R-B**	**PilF159-302 K167L-A**	**PilF159-302 K167L-B**
**MshEN-A**	0	0.290	1.728	2.318	1.737	1.727	1.950	1.931
**MshEN-B**	0.290	0	1.099	1.392	1.306	1.383	1.305	1.196
**PilF159-302 WT-A**	1.728	1.099	0	0.118	0.064	0.104	0.089	0.196
**PilF159-302 WT-B**	2.318	1.392	0.118	0	0.109	0.061	0.114	0.100
**PilF159-302 K167R-A**	1.737	1.306	0.064	0.109	0	0.089	0.065	0.168
**PilF159-302 K167R-B**	1.727	1.383	0.104	0.061	0.089	0	0.103	0.086
**PilF159-302 K167L-A**	1.950	1.305	0.089	0.114	0.065	0.103	0	0.145
**PilF159-302 K167L-B**	1.931	1.196	0.196	0.100	0.168	0.086	0.145	0

Supplementary Table 5

Primers, plasmids and PilF variants used for in-vivo studies.

**Primer**	**Sequence 5'-3'**	**Resulting plasmid**	**PilF variants**
**Q190E_for**	CTTGGTGGAAGAAGAGAAGACGGGGG	pET28a-*pilF*-Q190E	PilF-Q190E
**Q190E_rev**	GCCTCCTCCAGGGCCTCG	pET28a-*pilF*-Q190E	PilF-Q190E
**Q218E_for**	CTTGGCGGAGGAAAAGGGGCTGG	pET28a-*pilF*-Q218E	PilF-Q218E
**Q218E_rev**	GCCCGGTAAAGGGCCTCC	pET28a-*pilF*-Q218E	PilF-Q218E

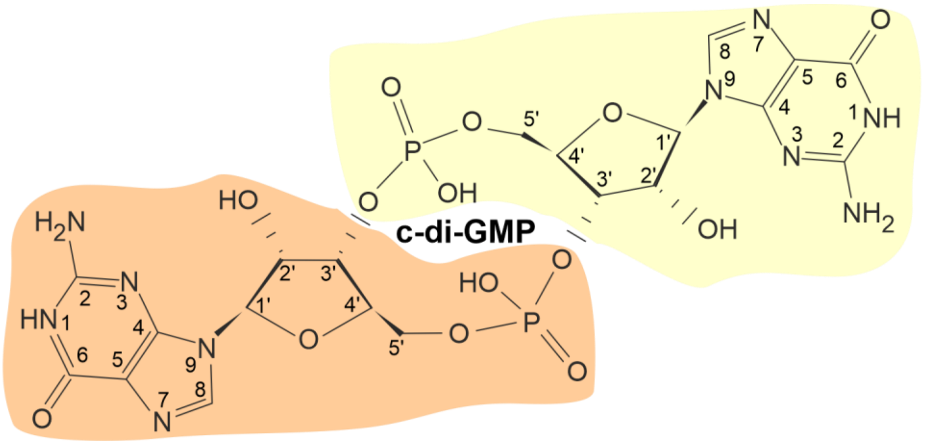

Supplementary Fig. S1: Chemical structure and atomic numbering scheme for c-di-GMP. The two GMP moieties are highlighted by color.

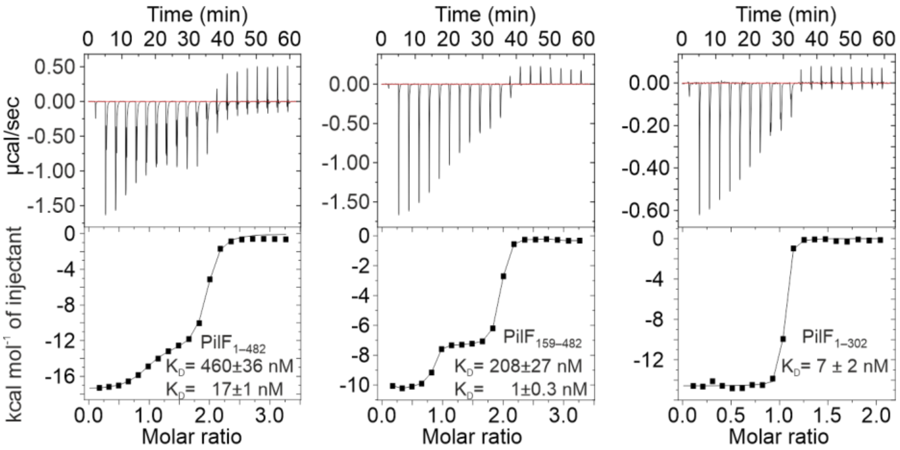

Supplementary Fig. S2: Isothermal titration calorimetry thermograms (top) and binding isotherms (bottom) for c-di-GMP binding to PilF$_{1\text{-}482}$ (left), PilF$_{159\text{-}482}$ (middle) and PilF$_{1\text{-}302}$ (right), respectively. The K_D's shown are averages of three independent titration experiments.

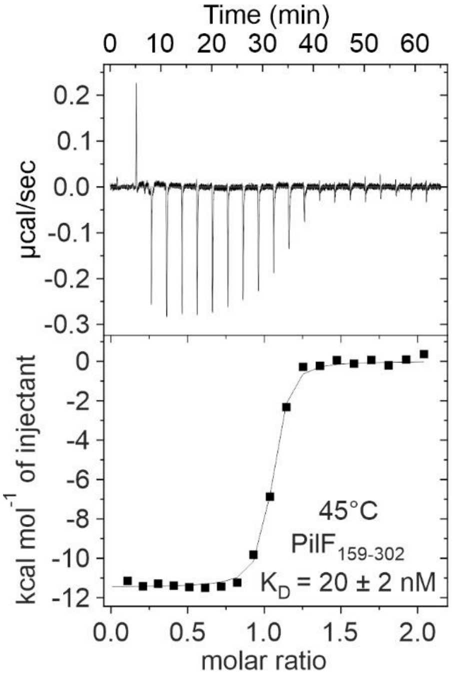

Supplementary Fig. S3: Isothermal titration calorimetry thermogram (top) and binding isotherm (bottom) for c-di-GMP binding to PilF$_{159\text{-}302}$ at 45 °C. The K_D is an average of three independent titration experiments.

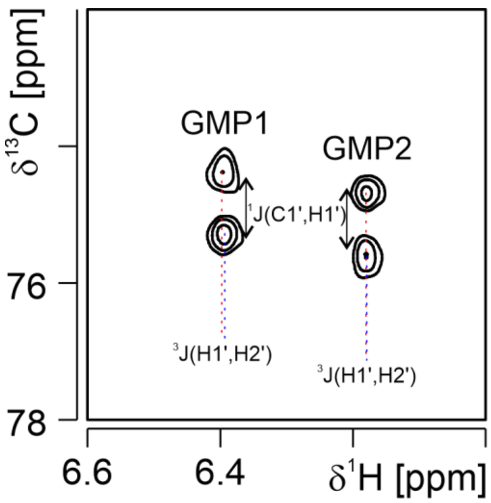

Supplementary Fig. S4: Forward-directed 2D-HCCH-TOCSY-CCH-E.COSY spectrum recorded for $^{13}C,^{15}N$-labelled c-di-GMP bound to $PilF_{159\text{-}302}$ to determine the ribose sugar pucker conformation. The blue and red dotted lines represent the peak maxima of the signals that are split in the proton dimension in relation to the H1',H2' $^3J_{HH}$ scalar coupling. For GMP1 a coupling constant of 1.5 Hz and for GMP2 a coupling constant of 0.4 Hz was measured.

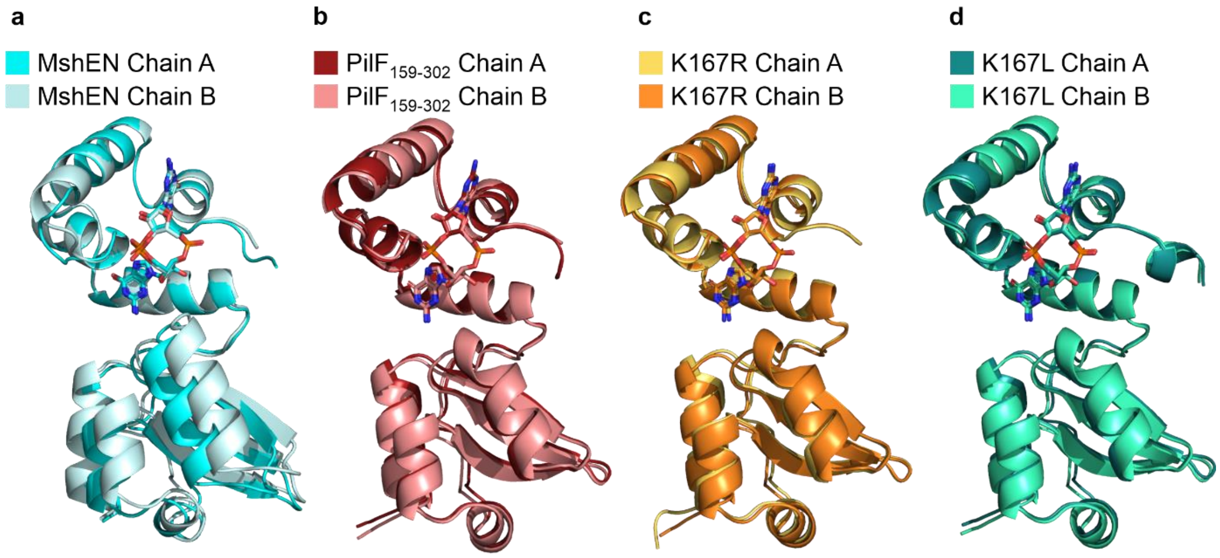

Supplementary Fig. S5: Superimposition of the N-terminal subdomains from the two polypeptide chains found in each unit cell of the X-ray structures of MshEN, PilF$_{159\text{-}302}$, PilF$_{159\text{-}302}$ K167R and PilF$_{159\text{-}302}$ K167L. ***a*** *MshEN chain A (cyan) and B (pale cyan) aligned by their N-terminal subdomains (RMSD = 0.212).* ***b*** *N-terminal subdomain superimposition of chain A (red) and B (salmon) of PilF$_{159\text{-}302}$ (RMSD = 0.6).* ***c*** *PilF$_{159\text{-}302}$ K167R chain A (yellow) and B (orange) aligned by their N-terminal subdomains (RMSD = 0.6).* ***d*** *PilF$_{159\text{-}302}$ K167L chain A (teal) and B (green cyan) aligned by their N-terminal subdomains (RMSD = 0.5).*

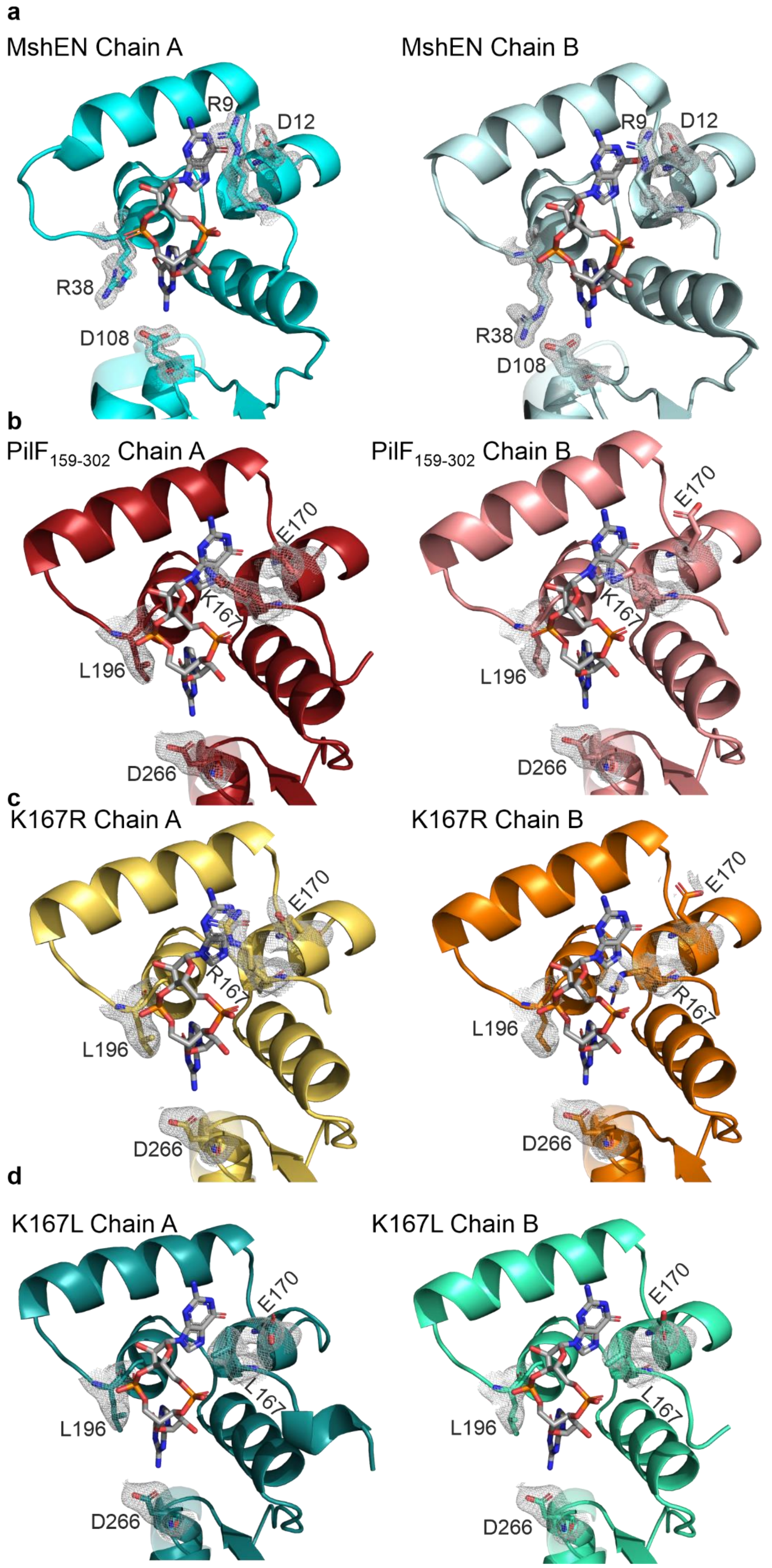
a
MshEN Chain A
MshEN Chain B
R9
D12
R38
D108
b
PilF159-302 Chain A
PilF159-302 Chain B
E170
K167
L196
D266
c
K167R Chain A
K167R Chain B
E170
R167
L196
D266
d
K167L Chain A
K167L Chain B
E170
L167
L196
D266

Supplementary Fig. S6: Comparison of the experimentally determined electron density for side chains important for ligand binding between chains A and B of the MshEN, $PilF_{159\text{-}302}$, $PilF_{159\text{-}302}$ K167R and $PilF_{159\text{-}302}$ K167L crystal structures. To highlight potential flexibility in the sidechain conformations the electron density of the involved residues is shown at 1σ as a grey mesh. ***a*** *Side by side comparison of chain A (left) and B (right) of the MshEN crystal structure. R9, D12, R38 and D108 are shown as sticks overlaid by their electron density to indicate confidence in sidechain placement.* ***b*** *Side by side comparison of chain A (left) and B (right) of the $PilF_{159\text{-}302}$ crystal structure. K167, E170, L196 and D266 are shown as sticks with their electron density overlaid. Interestingly, for E170 in chain B the electron density is not resolved at a contour level of 1σ.* ***c*** *Side by side comparison of chain A (left) and B (right) of the $PilF_{159\text{-}302}$ K167R crystal structure. R167, E170, L196 and D266 are shown as sticks with their electron density overlaid. Notably, in chain B electron density is weak for R167 and only resolved for its guanidinium group and not resolved for E170 at a contour level of 1σ.* ***d*** *Side by side comparison of chain A (left) and B (right) of the $PilF_{159\text{-}302}$ K167L crystal structure. L167, E170, L196 and D266 are shown as sticks with their electron density overlaid. Intriguingly, all sidechains in this construct are placed identical with electron densities resolved at 1σ even for E170.*

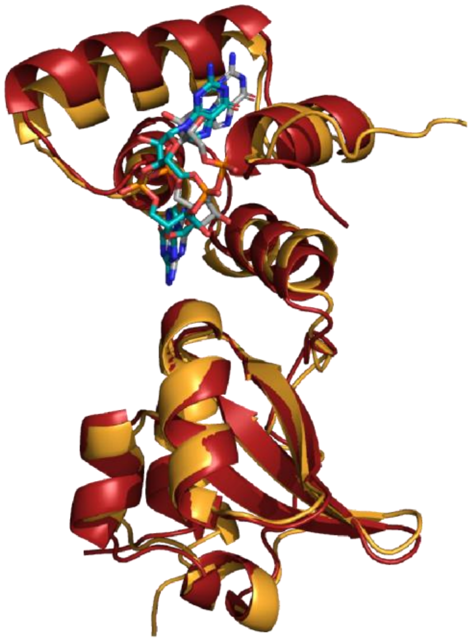

Supplementary Fig. S7: Overlay of c-di-GMP bound PilF$_{159\text{-}302}$ NMR- (yellow) and crystal structure (red, chain A) aligned with respect to the C-terminal subdomain.

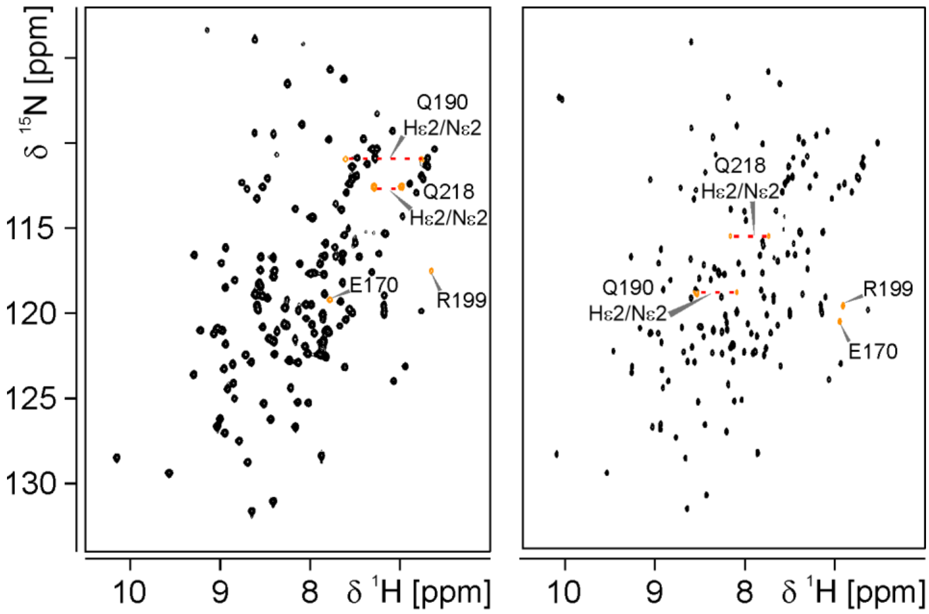

Supplementary Fig. S8: $^1H,^{15}N$-HSQC spectra of PilF$_{159\text{-}302}$ in the apo (left) and the holo (right) state. Key residues showing large chemical shift perturbations upon c-di-GMP binding are highlighted in orange. The dotted line in red connects the two resonances of the protons in the glutamine side chain amino groups of Q190 and Q218.

.

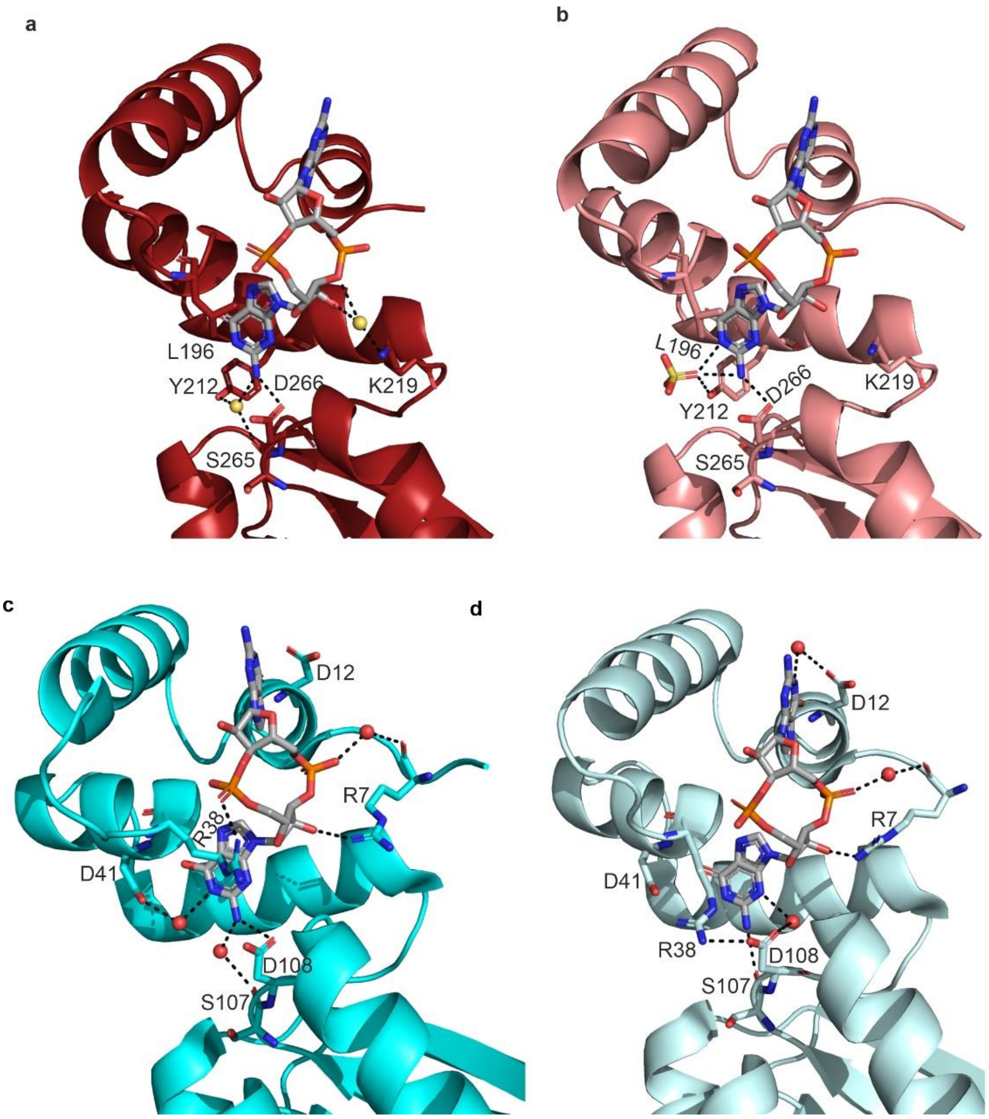

Supplementary Fig. S9: (a) Water mediated hydrogen bonds in ligand recognition in PilF$_{159\text{-}302}$. Important residues involved are shown as sticks, waters are shown as yellow spheres and hydrogen bonds are indicated by black dotted lines. Residues that are only involved in water mediated hydrogen bonds in one chain are always shown to highlight differences between the two chains. Notably, no water-mediated intermolecular hydrogen bonds are found in chain B (b) but a sulfate molecule (yellow sticks) is at the same position as a water in chain A. (c) Water mediated hydrogen bonds in ligand recognition in MshEN chain A and chain B (d).

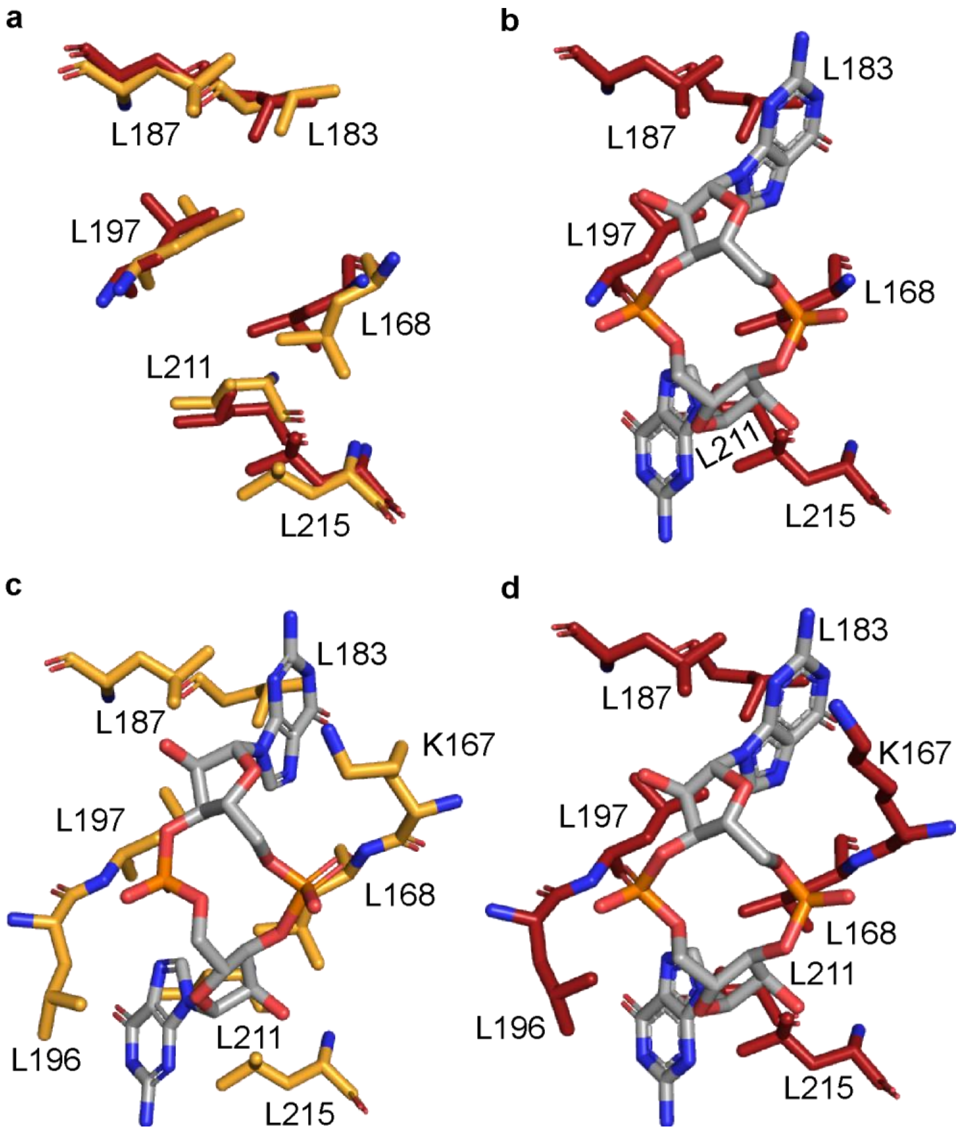

Supplementary Fig. S10: Comparison of stacking interactions of PilF$_{159\text{-}302}$ residues with c-di-GMP in the NMR and the crystal structure of the c-di-GMP bound PilF$_{159\text{-}302}$. ***a*** *Overlay of the triangular leucine clusters in the NMR- (yellow) and the X-ray- (red) structure. C-di-GMP is not depicted for reasons of clarity.* ***b*** *Stacking of the two triangular leucine clusters on c-di-GMP in the X-ray structure in the same orientation as in a).* ***c*** *All stacking interactions of PilF$_{159\text{-}302}$ with c-di-GMP (NMR-structure) including K167 and L196 which replace the two arginine residues in the MshEN derived consensus sequence.* ***d*** *All stacking interactions of PilF$_{159\text{-}302}$ with c-di-GMP (X-ray-structure).*

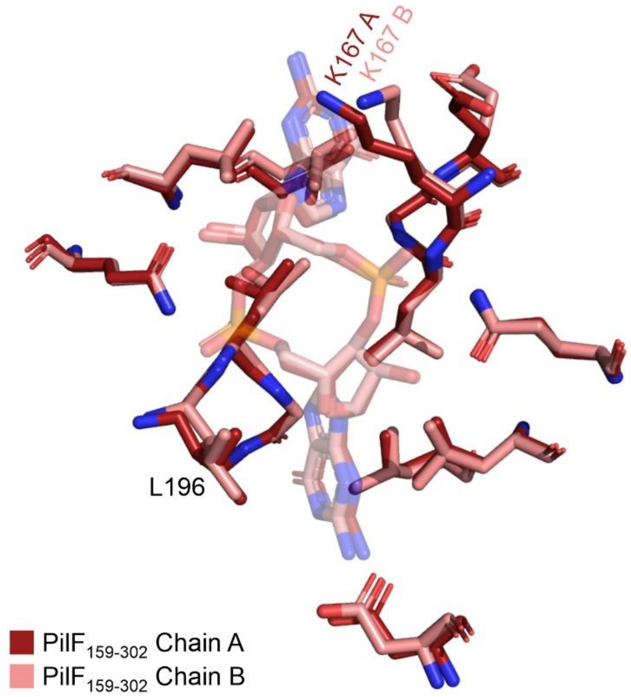

Supplementary Fig. S11: Comparison of the side chain conformations in the ligand binding pocket in PilF159-302 chains A and B.

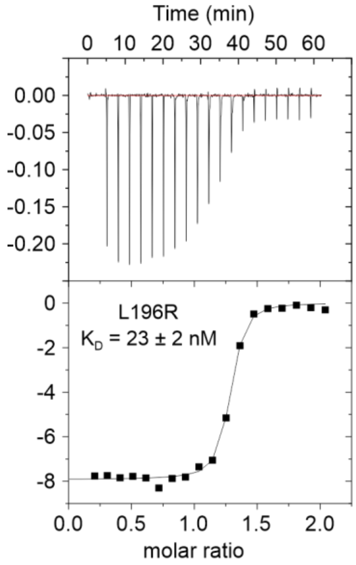

Supplementary Fig. S12: ITC thermogram (top) and binding isotherm (bottom) for c-di-GMP binding to the PilF$_{159\text{-}302}$ L196R variant.

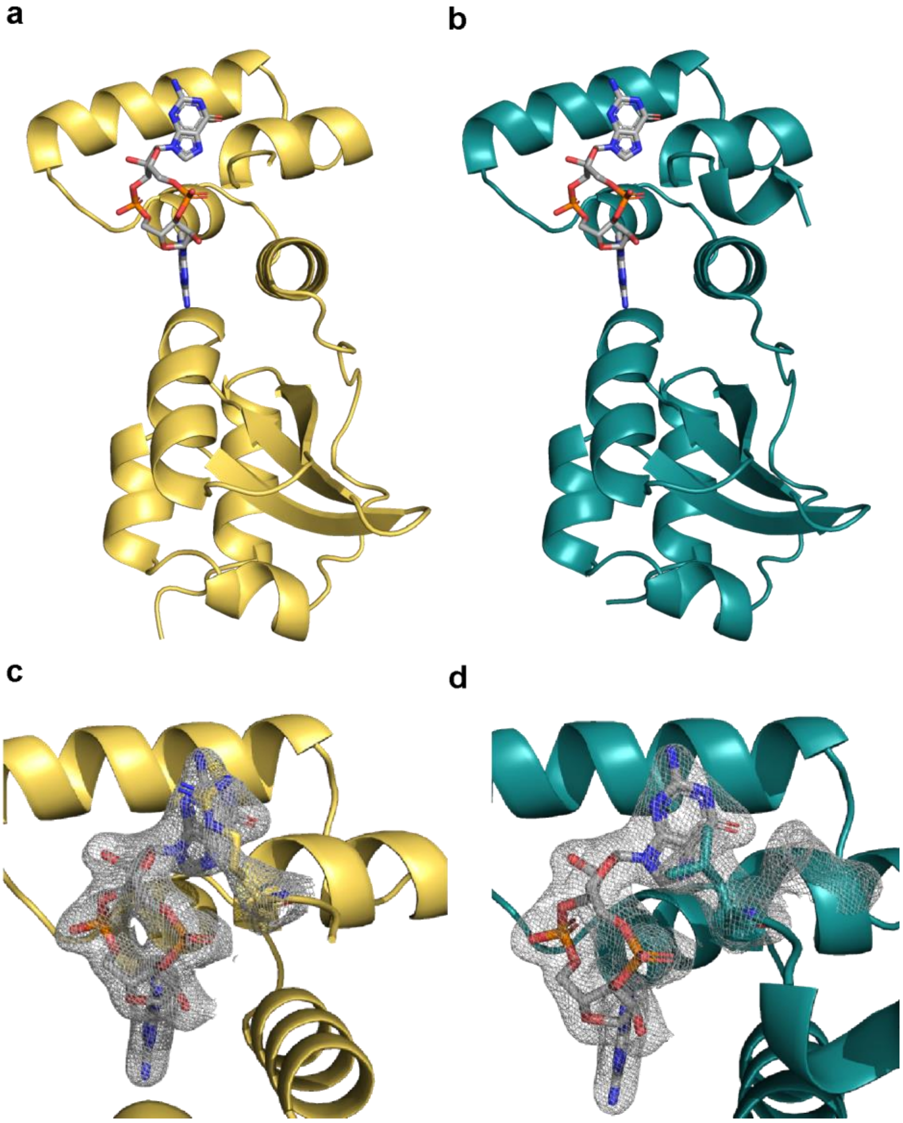

Supplementary Fig. S13: X-ray structures of PilF$_{159\text{-}302}$ K167R (yellow, left) and K167L mutants (teal, right). ***a*** *X-ray structure of PilF$_{159\text{-}302}$ K167R.* ***b*** *X-ray structure of PilF$_{159\text{-}302}$ K167L.* ***c*** *Close-up on R167 stacking onto the guanine base of c-di-GMP in the PilF PilF$_{159\text{-}302}$ K167R mutant. The F_o-F_c electron densities of bound c-di-GMP and R167 are drawn at the 1.0σ level.* ***d*** *Close-up on L167 stacking onto the guanine base of c-di-GMP in the PilF PilF$_{159\text{-}302}$ K167L mutant. The F_o-F_c electron densities of bound c-di-GMP and L167 are drawn at 1.0σ level. In all panels chain A of the respective crystal structure is shown.*

.

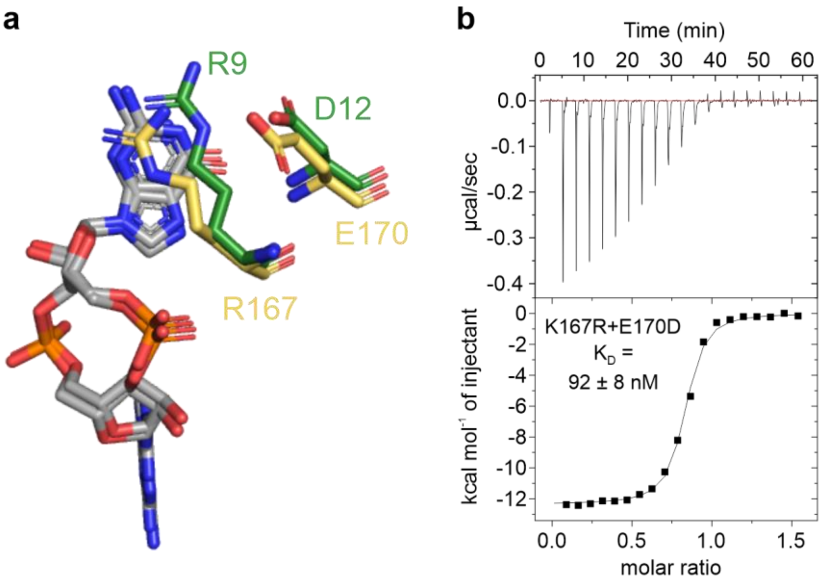

Supplementary Fig. S14: ***a*** *Close-up on the cation-π-stacking interaction of MshEN chain A (green) and the K167R PilF$_{159-302}$ mutant chain A (yellow).* ***b*** *ITC thermogram (top) and binding isotherm (bottom) for c-di-GMP binding to the PilF$_{159-302}$ K167R-E170D variant.*

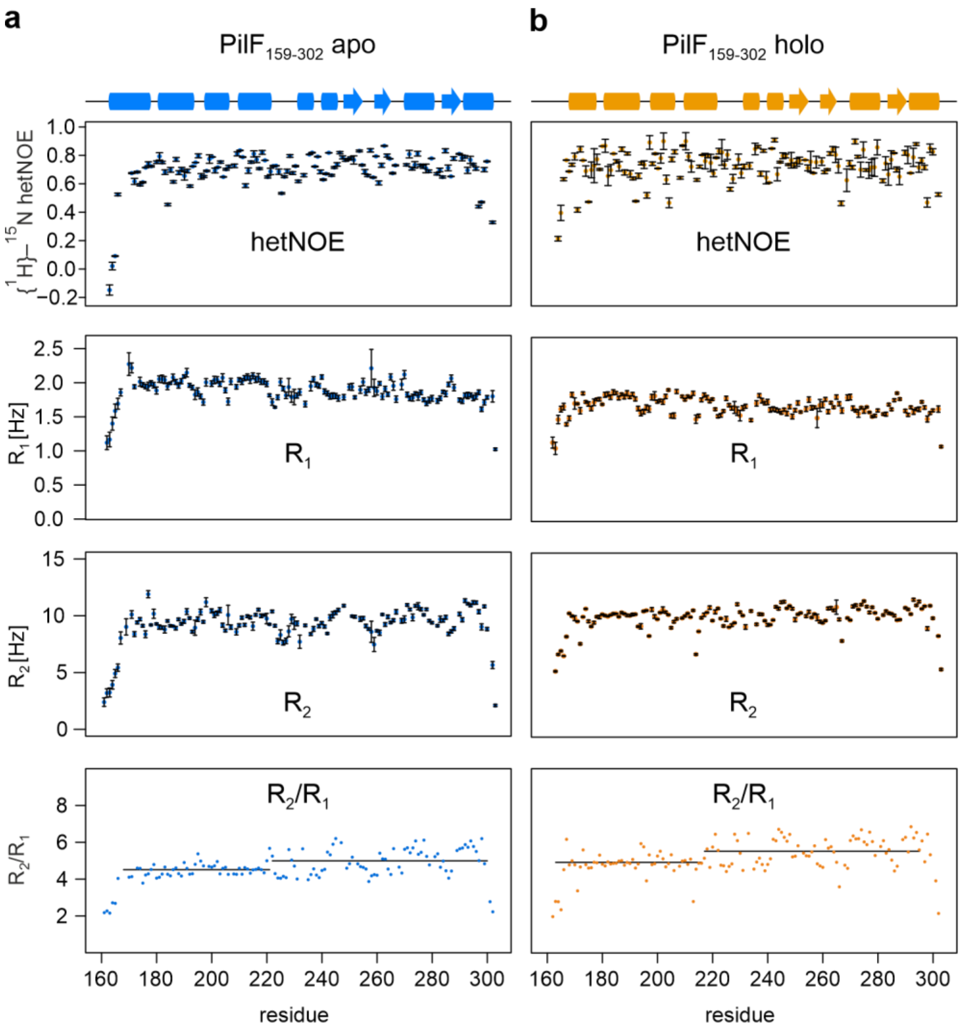

*Supplementary Fig. S15: Protein dynamics of PilF$_{159\text{-}302}$ in the apo- and the holo-state. {^{1}H}-^{15}N hetNOE values, longitudinal (R_1) and transversal (R_2) relaxation rates of PilF$_{159\text{-}302}$ in the apo- (**a**) and the holo- (**b**) state plotted against the sequence. The black bars in the bottom diagrams represent the median of the R_1/R_2 ratio of the N- and C-terminal subdomains.*

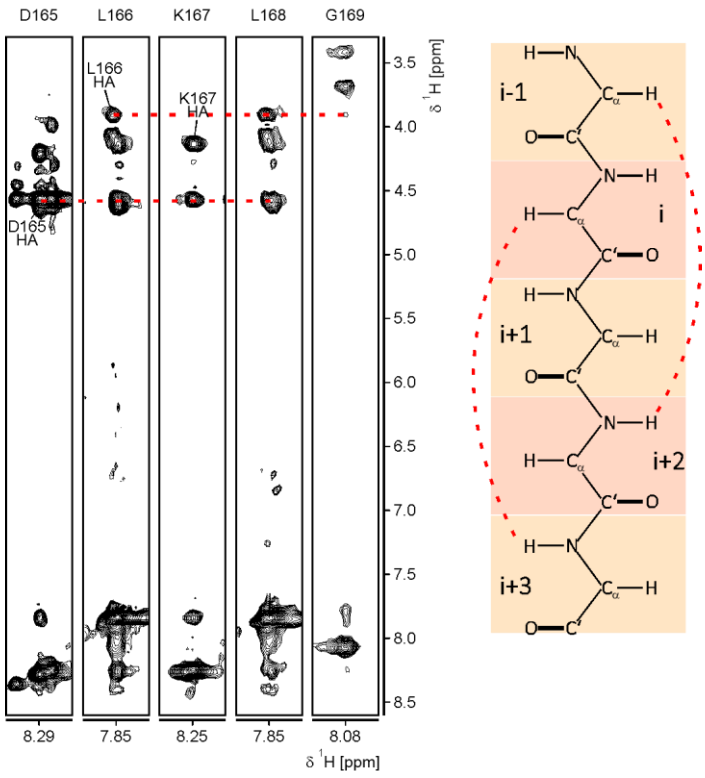

Supplementary Fig. S16: NOE-pattern of PilF$_{159\text{-}302}$ residues D165-G169 in the apo-state. The typical NOE-pattern for an α-helix is shown on the right.

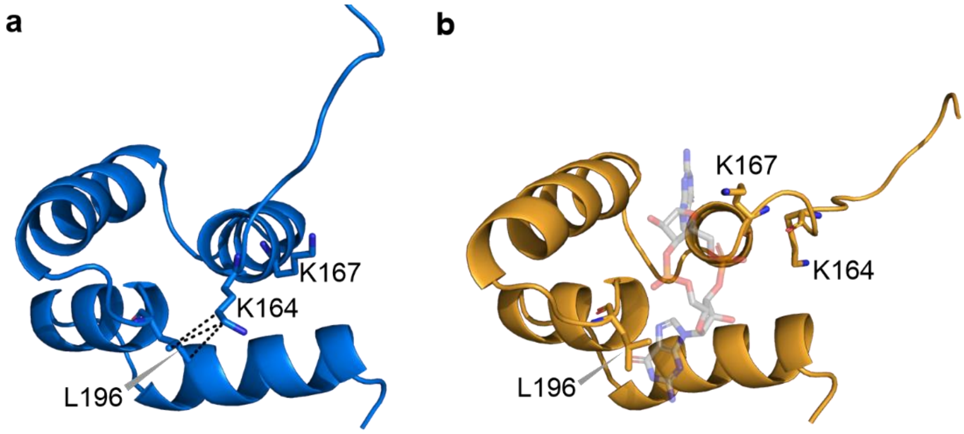

Supplementary Fig. S17: Comparison of the N-terminal subdomains of the NMR solution structures of PilF$_{159\text{-}302}$ in the apo and holo state focused changes in on intramolecular interactions upon ligand binding. ***a*** *NMR solution structure of the PilF$_{159\text{-}302}$ apo state focused on the N-terminal subdomain. K164, K167 and L196 are shown as sticks and hydrophobic interactions between K164 of helix 1 and L196 of helix 3 are indicated as black dotted lines.* ***b*** *NMR solution structure of the PilF$_{159\text{-}302}$ holo state focused on the N-terminal subdomain. c-di-GMP is shown in a transparent stick presentation. Notably, K164 and L196 are separated upon ligand recognition, the helical turn containing K164 in the apo state is unfolded, and K167 is involved in a hydrophobic stacking interaction with one guanine base.*

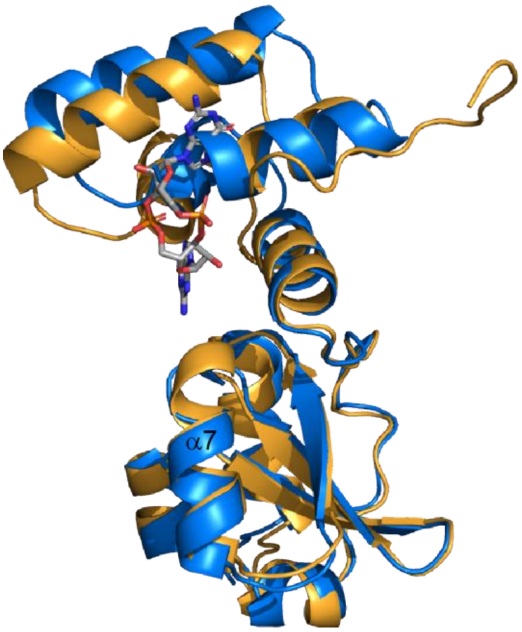

Supplementary Fig. S18: Overlay of PilF$_{159\text{-}302}$ in the apo- (blue) and in the holo-state (yellow) aligned with respect to the C-terminal subdomain.

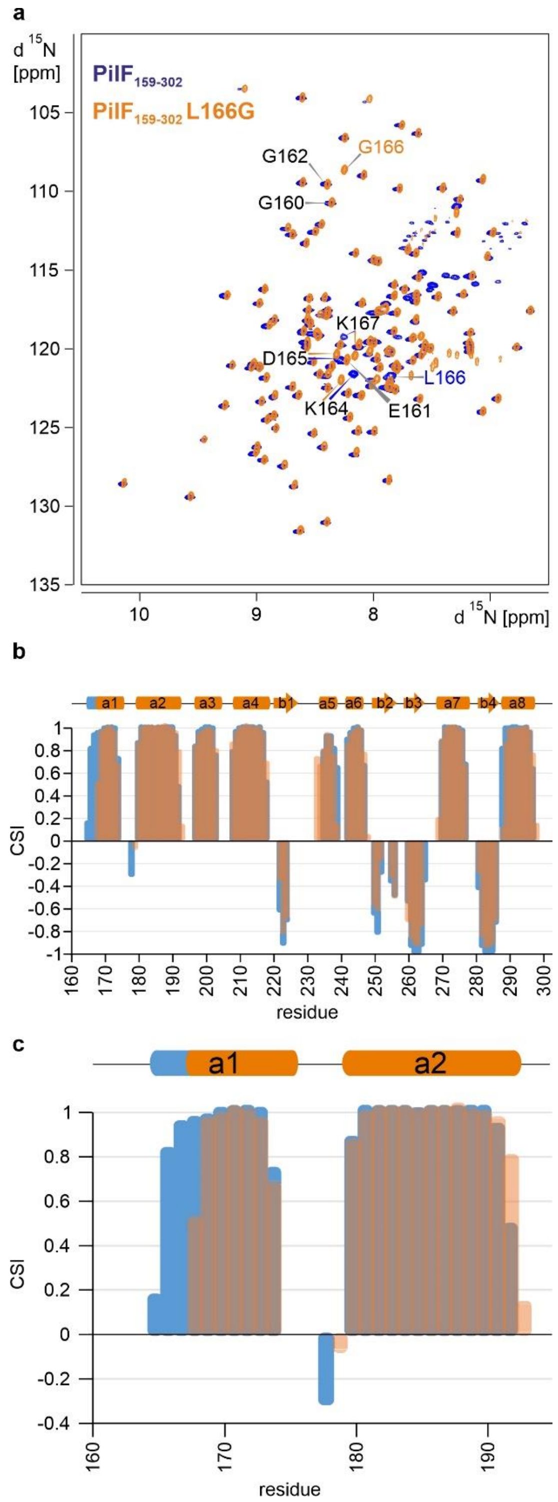

Supplementary Fig. S19: Helix α1 is shortened in the apo state of the L166G mutant. (a) Overlay of the ^{1}H,^{15}N-HSQC-spectra of the WT and the L166G mutant with assignments for the N-terminal residues indicated. (b) Comparison of the CSI derived from the backbone resonance assignments for the mutant (orange) and the WT protein (blue) for the full protein and (c) the first two α-helices.

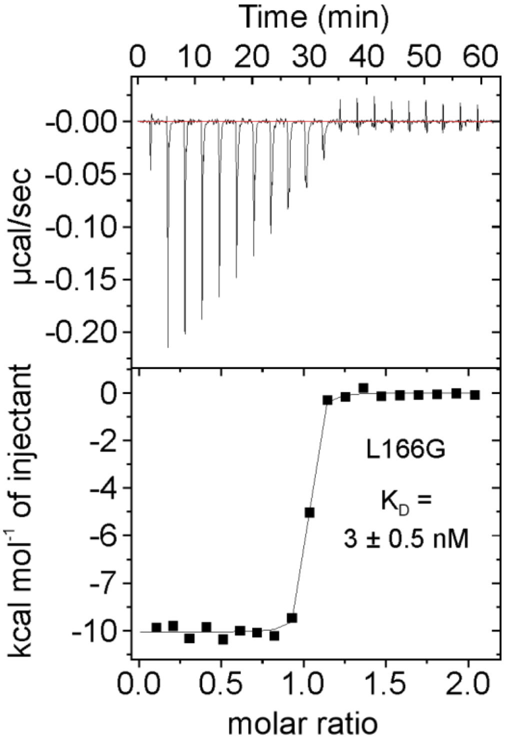

Supplementary Fig. S20: ITC thermogram (top) and binding isotherm (bottom) for c-di-GMP binding to PilF$_{159-302}$ L166G.

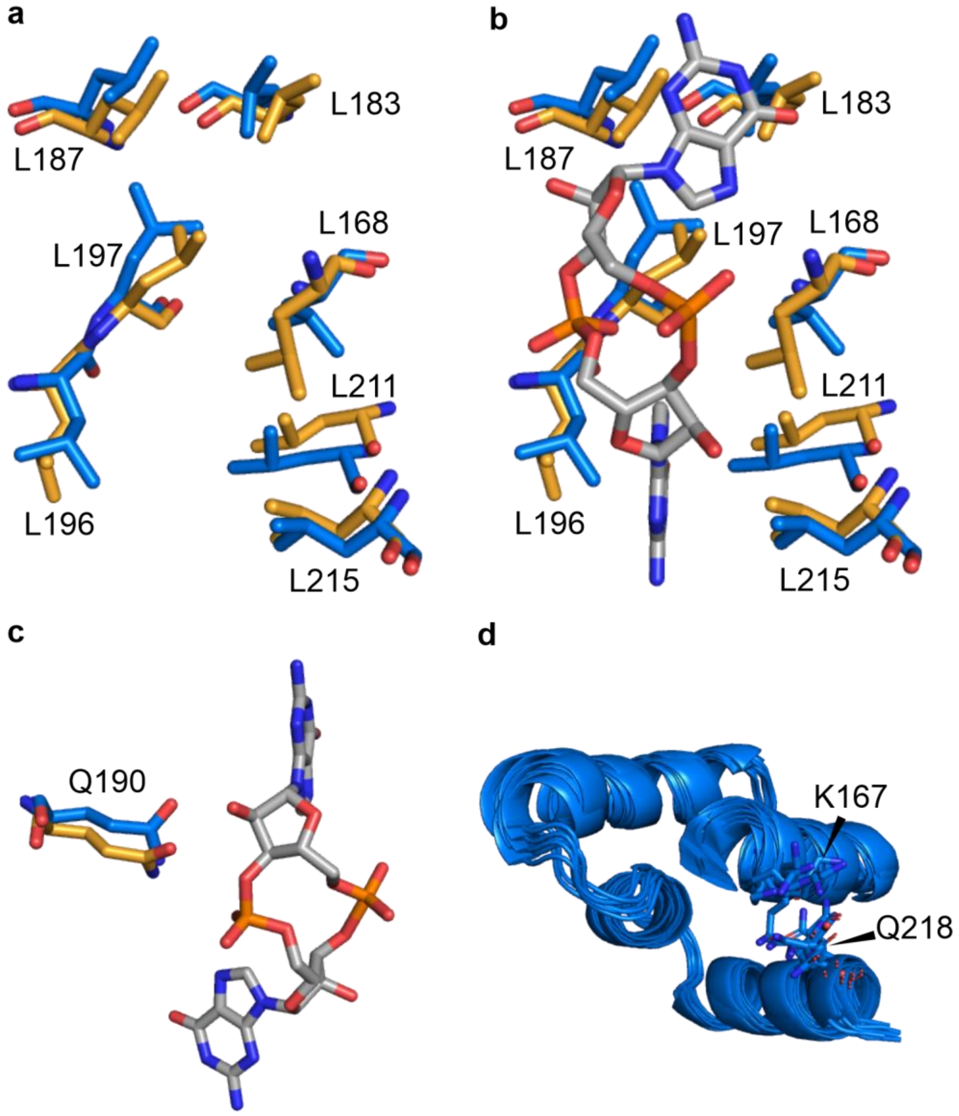

Supplementary Fig. S21: Side chain orientations of key residues involved in c-di-GMP binding in the PilF$_{159\text{-}302}$ apo-state (blue) compared to the c-di-GMP bound state (orange) in the respective NMR solution structures. ***a*** *Overlay of triangular leucine clusters in the apo- and the holo-state (c-di-GMP is not shown for simplicity).* ***b*** *Overlay of leucine triangles in the apo- and the holo-state with c-di-GMP.* ***c*** *Q190 in the apo- and the holo-state is oriented towards the ribose-phosphate backbone of the c-di-GMP molecule in the holo-state NMR structure* ***d.*** *N-terminal subdomain structure bundle for the ten best NMR-structures in the apo-state with the side chains of K167 and Q218 in stick presentation.*

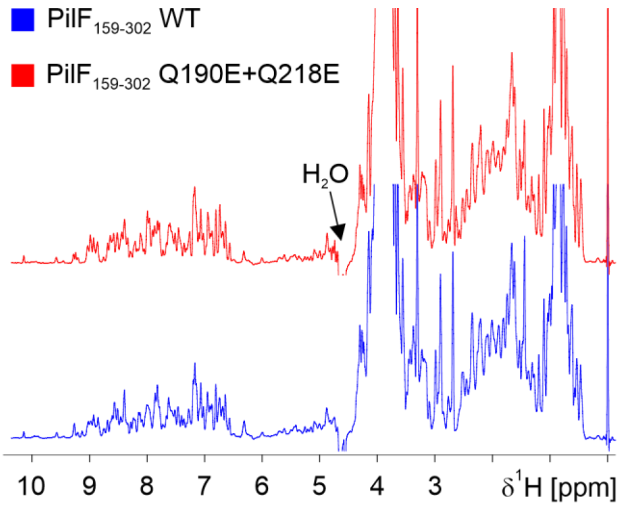

Supplementary Fig. S22: 1D-^{1}H proton spectra of WT-PilF$_{159\text{-}302}$ (apo, blue) and the double mutant PilF$_{159\text{-}302}$ Q190E/Q218E (apo, red) showing that both proteins are well folded indicating that the Q190E/Q218E double mutation has no effect on the structural integrity of the protein.
